# *Trichoderma*-Based Biopreparation with Prebiotics Supplementation for the Naturalization of Raspberry Plant Rhizosphere

**DOI:** 10.3390/ijms22126356

**Published:** 2021-06-14

**Authors:** Karolina Oszust, Michał Pylak, Magdalena Frąc

**Affiliations:** Institute of Agrophysics, Polish Academy of Sciences, Doświadczalna 4, 20-290 Lublin, Poland; m.pylak@ipan.lublin.pl (M.P.); m.frac@ipan.lublin.pl (M.F.)

**Keywords:** agroecology, naturalization, raspberry rhizosphere, biopreparation, *Trichoderma*, antagonism, prebiotics, hydrolytic enzymes

## Abstract

The number of raspberry plants dying from a sudden outbreak of gray mold, verticillium wilt, anthracnosis, and phytophthora infection has increased in recent times, leading to crop failure. The plants suffer tissue collapse and black roots, symptoms similar to a *Botrytis*–*Verticillium–Colletotrichum*–*Phytophthora* disease complex. A sizeable number of fungal isolates were acquired from the root and rhizosphere samples of wild raspberries from different locations. Subsequent in vitro tests revealed that a core consortium of 11 isolates of selected *Trichoderma* spp. was the most essential element for reducing in phytopathogen expansion. For this purpose, isolates were characterized by the efficiency of their antagonistic properties against *Botrytis*, *Verticillium*, *Colletotrichum* and *Phytophthora* isolates and with hydrolytic properties accelerating the decomposition of organic matter in the soil and thus making nutrients available to plants. Prebiotic additive supplementation with a mixture of adonitol, arabitol, erythritol, mannitol, sorbitol, and adenosine was proven in a laboratory experiment to be efficient in stimulating the growth of *Trichoderma* isolates. Through an in vivo pathosystem experiment, different raspberry naturalization-protection strategies (root inoculations and watering with native *Trichoderma* isolates, applied separately or simultaneously) were tested under controlled phytotron conditions. The experimental application of phytopathogens attenuated raspberry plant and soil properties, while *Trichoderma* consortium incorporation exhibited a certain trend of improving these features in terms of a short-term response, depending on the pathosystem and naturalization strategy. What is more, a laboratory-scale development of a biopreparation for the naturalization of the raspberry rhizosphere based on the *Trichoderma* consortium was proposed in the context of two application scenarios. The first was a ready-to-use formulation to be introduced while planting (pellets, gel). The second was a variant to be applied with naturalizing watering (soluble powder).

## 1. Introduction

Raspberries are certainly valuable fruit. They have health-promoting, antioxidant and anti-inflammatory properties, and at the same time, they are juicy and aromatic, which is why they are such a readily purchased commodity. Therefore, the cultivation of raspberries plays a very important role in the horticultural production of many countries around the world. According to FAO reports [1], the world’s top raspberry producers in recent years were Russia, the United States, and Poland, followed by Mexico and Serbia. Moreover, raspberry harvests show a clear upward trend [2].

Unfortunately, the phytopathogens of raspberries, which belong to the genus of *Botrytis* sp., *Verticillium* sp. *Colletotrichum* sp. or *Phytophthora* sp. for the most part cause enormous losses in the production of these fruit. These pathogens lead to diseases such as gray mold, verticillium wilt, anthracnosis, and phytophthora infection in soft fruit production, respectively, which lowers product quality [3]. Due to this, there is an urgent need to develop effective biopreparations, which can stimulate the growth and resistance of raspberry plants, especially through the natural mechanisms of competition among microbes, including invasive fungal and fungal-like pathogens [4].

Increasing interest in organic farming and especially in the organic production of raspberry fruit is considered to be a factor encouraging farmers to develop new ecological solutions in plant biostimulation and protection by using natural microbe-based products. What is more, European Commission Regulation no. 834/2007 [5] states that the use of chemical pesticides must be limited to an absolute minimum, and furthermore that farmers are encouraged to use substances of a natural origin. Additionally, the EU Biodiversity Strategy for 2030 [6], which was introduced very recently, sets forth the importance of biodiversity in all environments [7]. It includes the policy of making agricultural land more natural. This strategy has the intention of not only reducing the use of chemicals by at least 50% but also of increasing the area used for organic farming by up to 25% of all land being used for agriculture in the coming decade. What is more, organic farmers are obliged to refrain from using any commercially available synthetic pesticides and fertilizers. As a consequence, their yields may be lowered and their costs may be increased. Therefore, one of the proposed strategies for agroecology could be the application of microbes used as drivers of soil services [8] which includes the formation of structured, complex interconnected microbial networks which can act as mediators between the plant and microbiomes within the framework of being biocontrol agents [9].

In particular, *Trichoderma* fungi are widely regarded as effective and safe, due to their use as natural biopreparations dedicated to green practices for the production of horticultural crops [10]. They are widely known as the silent workers of the plant rhizosphere [11]. The importance of the interaction between *Trichoderma* spp., soilborne pathogens and the plant is often emphasized in the literature. Fungi of the genus *Trichoderma* also act as biocontrol factors, by manifesting several typical mechanisms, such as the production of compounds that inhibit the development of pathogens, mycoparasitism, the inactivation of pathogen enzymes, the induction of plant systemic immunity or competition for nutrients, i.e., for living space by creating mycelial biomass. *Trichoderma* fungi are widely known to grow very rapidly and are considered to be aggressive competitors against pathogens [12,13,14,15].

The ability of *Trichoderma* spp. to effectively decompose dead organic matter results from the production of several lytic enzymes [16] and among other benefits this contributes to the improvement of the physicochemical properties of the soil and thereby improves its quality [17]. The fungi of the *Trichoderma* genus, as a result of their hydrolytic activity, release macro- and microelements from organic matter into the soil solution which are used by plants [18]. Thus, strains of *Trichoderma* are often used as a component of biopreparations [19].

The state of the art in this area describes fungal representatives belonging to the *Trichoderma* genus which exhibit antagonistic properties against many fungal plant pathogens. Some of the world’s most significant examples of studies and developments in this area from the last decade were summarized recently [20]. As outlined in the presentation, the most frequently conducted research and patents, which were sought and obtained, concern *Trichoderma*-based biopreparations used for suppressing phytopathogens belonging to the *Pythium*, *Phytophthora*, *Penicillium*, *Fusarium*, and *Sclerotium* genera in many horticultural and agricultural plants. However, to date, there are almost no solutions based on the use of *Trichoderma* spp., as biostimulants of plants and microbes which at the same time control phytopathogens belonging to the genus *Botrytis*, *Verticillium*, *Colletotrichum*, and *Phytophthora* in raspberry plantations. What is more, raspberry plantations have been rather overlooked as a potential target for microbial-based biopreparations, although there is no doubt that this is a significant marketplace for ecological solutions [2]. However, this issue has been addressed quite recently [21].

In the literature on the subject, for the development of biopreparations, various matrices and carriers are used [22]. Authors expertly compare carriers, their types, their formation and inoculation, and finally their role in the plant agrosystem, and simultaneously point out the need for meticulous planning to match the appropriate formulation to the correct predesigned method of its application.

By definition, the newly developed biopreparation should contain probiotic microorganisms with the activity of improving the microbiological quality of the soil as effectively as possible. In order to bring the desired result a step closer, a concept concerning naturalization, which is also called the biotization phenomena, was developed. It assumes that the source of the beneficial/probiotic microorganisms such as natural, healthy habitats, unchanged by synthetic chemical treatment (or their natural equivalents) may bring about the expected functionality of the final bioproducts in terms of quality. As previously stated, the microbial strains obtained from such environments show activity that supports the growth and functioning of particular plants or/and manifest antagonistic properties [23,24]. For planted raspberries, the corresponding natural environment includes wild raspberry plants [2].

Plant-associated probiotic microorganisms such as *Trichoderma* spp. are naturally occurring microorganisms that enhance the growth of the host plants including increases in yield and may suppress diseases when applied in adequate amounts [25]. Probiotic microorganisms have become increasingly popular during the last two decades as a result of the continuously expanding scientific evidence pointing to their beneficial effects not only on the well-being of plants but also on human [26] and animal [27] health. Going forward, prebiotics is also a significant topic in human and animal nutrition at present. However, at present, there are few reports in the literature regarding the supplementation of biopreparations with additives that improve the food competitiveness of beneficial microbes which act against plant pathogens. The initial and preliminary research based on Biolog^®^ (Hayward, Canada) microplates, suggesting specific prebiotic additives for the future application into *Trichoderma*-based biopreparations was published [4]. Regarding the touchstones mentioned above, the goals and hypothesis of the presented study were as follows.

Firstly, we aimed to compose a fungal consortium for the biostimulation of plant growth and resistance, including effectiveness against the pathogens *Botrytis* spp. (including *B. cinerea*), *Verticillium* spp. *Colletotrichum* spp. (including *C. acutatum*) and *Phytophthora* spp., as well as efficiency in terms of the circulation of organic matter. Therefore, we hypothesized that the rhizosphere, rhizoplane, and roots of wild raspberries are valuable sources of beneficial fungal isolates. We followed these assumptions by acting aimed at obtaining an isolate collection of wild raspberries, selecting particular isolates that do not interact antagonistically with each other, but manifest strong antagonistic properties against the aforementioned pathogens and demonstrate reasonable hydrolytic properties.

Secondly, we seek to quantify the positive influence of the carefully selected *Trichoderma*-based consortium on raspberry plants and soil-associated properties. Thus, a raspberry pot experiment was set up, including *Botrytis*, *Verticillium*, *Colletotrichum* and *Phytophthora* pathosystems, and different *Trichoderma*-naturalization strategies, including root inoculations and watering, as well as treatments without pathogen contamination. We considered the differentiated reaction in raspberry biometrics, as well as the diverse response of selected plant macro- and micronutrient content, depending on the pathosystem and naturalization strategy applied.

Thirdly, concerning the above, a unique step-by-step development of the biopreparation targeted at raspberry naturalization was described. This proposal included an assortment of *Trichoderma* spp. culture medium compositions, considering the differences between individual isolates in terms of nutritional requirements, carriers, and finally prebiotic supplement addition. As the motivation for conducting the present study was to develop the formulation concepts towards tolerably different practical application methods of the *Trichoderma*-based consortium, several hydrocolloid gelling ingredients and pellet compositions were tested.

## 2. Results

### 2.1. Isolates Description and Characterization

#### 2.1.1. Antagonists Selection

There were 159 fungal isolates acquired in total from the wild raspberry, including 123 isolates from the rhizosphere and 36 from the root material (Table 1). The isolation was intended as the very first step in the search for new beneficial fungal isolates of wild raspberries for biostimulation, including plant growth and resistance, as well as the biocontrol of phytopathogens.

**Figure 1:**
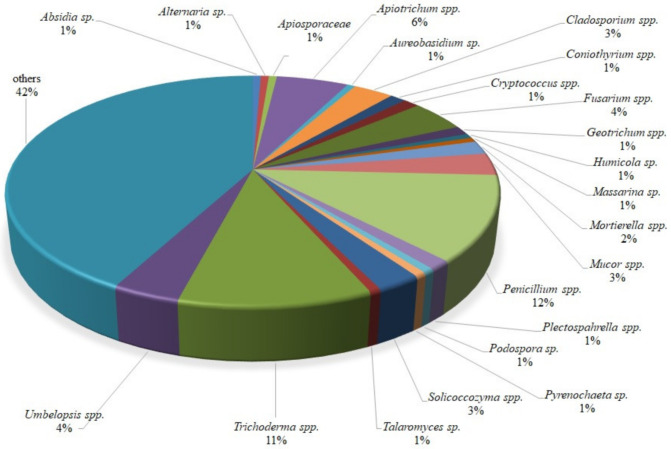
The percentage of fungal individual genera isolated from wild raspberries following identification based on the ITS or D2 LSU rDNA gene fragment.

Identification showed that one isolate representative of the following genera, *Absidia*, *Alternaria*, *Apiosporaceae*, *Aureobasidium*, *Humicola*, *Massarina*, *Podospora*, *Pyrenochaeta* and *Talaromyces*, was isolated. More than one isolate was acquired for the following genera: *Apiotrichum* (9), *Cladosporium* (5), *Coniothyrium* (2), *Cryptococcus* (2), *Fusarium* (7), *Geotrichum* (2), *Mortierella* (3), *Mucor* (5), *Penicillium* (19), *Plectosphaerella* (2), *Solicoccozyma* (4), *Umbelopsis* (6), *Trichoderma* (17), and others (67). The composition (%) of isolates obtained from wild raspberries is presented in Figure 1.

The 94 isolates listed in Table S1 from a total of 159 were regarded as potentially beneficial and following their selection they were screened for their antagonistic properties (growth and sporulation inhibition) against phytopathogenic fungi. The following phytopathogenic organisms, *Colletotrichum* spp. (G172/18, G371/18, G166/18), *Verticillium* spp. (G293/18, G296/18, G297/18), *Phytophthora* spp. (G368/18, G373/18, G369/18) and *Botrytis* spp. (G275/18, G277/18, G276/18), were taken into consideration as representatives.

Fungal isolates G59/18, G62/18, G63/18, G64/18, G67/18, G70/18, G74/18, G75/18, G77/18, G78/18, G79/18, G80/18, G109/18, G113/18, G378/18, G387/18, G393/18, G60/18, G61/18, G65/18, G66/18, G68/18, G69/18, G71/18, G72/18, G73/18, G76/18, G97/18, G118/18, G119/18, G388/18, and G392/18 were observed to reveal either very good or good antagonistic properties against *Colletotrichum* spp., which constituted 20.1% of the organisms collected from wild raspberry.

Very good or excellent activity against *Verticillium* spp. was noted for isolates G60/18, G61/18, G63/18, G64/18, G65/18, G66/18, G67/18, G68/18, G69/18, G71/18, G73/18, G74/18, G75/18, G76/18, G77/18, G78/18, G79/18, G80/18, G109/18, G378/18, G398/18, G72/18, and G387/18. They accounted for 14.5% of the wild raspberry fungal collection.

As for prompt antagonistic abilities against *Phytophthora* spp., 30.8% of the tested wild raspberry fungal isolates fit into this category. These were the following: G60/18, G62/18, G63/18, G73/18, G75/18, G79/18, G86/18, G112/18, G113/18, G140/18, G155/18, G159/18, G381/18, G386/18, G395/18, G39718, G59/18, G61/18, G64/18, G65/18, G66/18, G67/18, G68/18, G69/18, G71/18, G72/18, G74/18, G76/18, G77/18, G78/18, G87/18, G92/18, G109/18, 118/18, G375/18, G378/18, G383/18, G384/18, G385/18, G387/18, G388/18, G389/18, G390/18, G391/18, G392/18, G393/18, G398/18, G399/18, G70/18, G90/18, G119/18, and G396/18.

Isolates G59/18, G60/18, G61/18, G62/18, G63/18, G64/18, G68/18, G69/18, G72/18, G73/18, G74/18, G75/18, G80/18 G90/18, G113/18, G114/18, G119/18, G379/18, G383/18, G385/18, G387/18, G388/18, G391/18, G126/18, and G139/18 were observed to show very good or excellent activity against *Botrytis* spp. This represented 15.7% of the collected wild raspberry fungal isolates.

**Figure 2:**
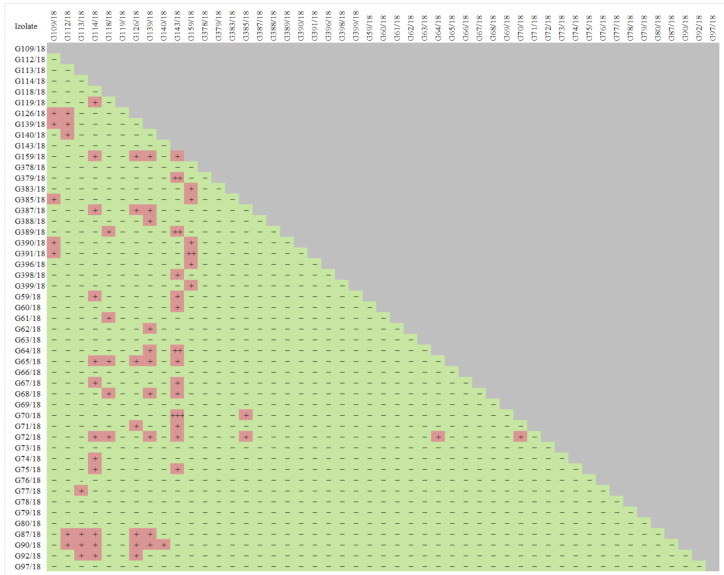
The antagonistic properties of wild raspberry fungal isolates against each other resulting in growth and/or sporulation inhibition in an experiment on Petri dishes. Abbreviations: the antagonism abilities rating was used. Abbreviations: “+++, ++, +, −” meaning excellent, very good, good, and no response, respectively.

Based on the results of the screening experiment (Table S1), the 49 isolates showing a relatively high potential to act against the tested pathogens *Colletotrichum* spp., *Verticillium* spp., *Phytophthora* spp., *Botrytis* spp. were preselected. These were isolates, which were proven to act against particular pathogens based on testing and also, they were previously known for additional good (but not so prominent) antagonistic abilities. The list of chosen isolates for further analyses is shown in Figure 2. Furthermore, the test results of the antagonistic properties of the selected isolates against each other were summarized since it is important to compose biopreparations with a microbial consortium that not only displays biocontrol activity against pathogens but also includes isolates that do not interact negatively with each other, which minimizes the possibility of low activity in the target environment.

In Table 2. the antagonistic properties of *Trichoderma* isolates against phytopathogens are presented. 

An antagonistic effect was noted among the tested isolates. G126/18, G139/18, G159/18, G387, G391/18, G370/18, G72/18, G87/18, G90/18, G92/18 G109/18, G64/18, G65/18, G67/18 are just the examples of isolates that interact. However, careful cross-testing of all isolates allowed for 11 isolates that do not interact negatively (Figure 2) to be chosen, but at the same time, they show a negative influence on pathogen development, resulting in its growth and/or sporulation inhibition (Table 2). These were as follows: G61/18, G63/18, G64/18, G65/18, G67/18, G69/18, G70/18, G78/18, G109/18, G379/18, and G398/18. The selection process was very careful, so as to not choose isolates that are recognized to be potential pathogens and omit those isolates with uncertain identification.

**Figure 3:**
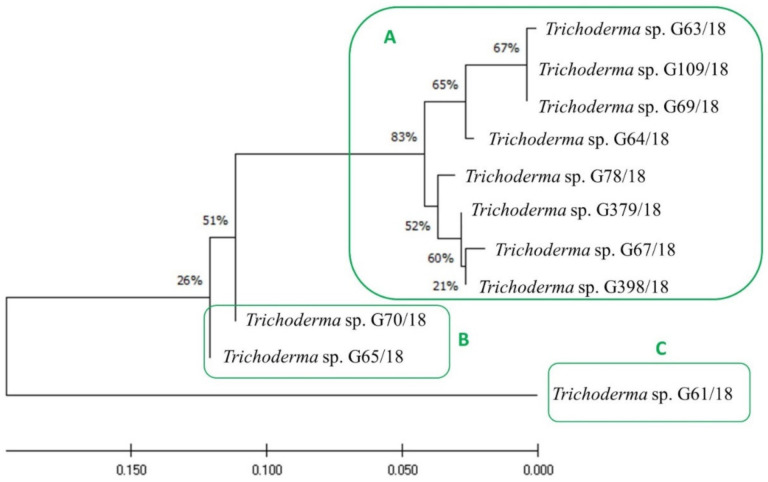
Evolutionary analysis of *Trichoderma* isolates using the maximum likelihood method. The evolutionary history was inferred by using the maximum likelihood method and the Tamura–Nei model. The tree with the highest log likelihood (−753.92) is shown. The percentage of trees in which the associated taxa are clustered together is shown next to the branches. The initial tree for the heuristic search was obtained automatically by applying the Neighbor-Join and BioNJ algorithms to a matrix of pairwise distances estimated using the Tamura–Nei model and then selecting the topology with a superior log-likelihood value. The tree is drawn to scale, with branch lengths measured in the number of substitutions per site. The proportion of sites where at least one unambiguous base is present in at least one sequence for each descendent clade is shown next to each internal node in the tree. This analysis involved 11 nucleotide sequences. There were a total of 346 positions in the final dataset. Evolutionary analyses were conducted in MEGA X (University Park, Pennsylvania, USA). (**A**–**C**) mean clustering isolates.

Those 11 isolates were described as belonging to the *Trichoderma* genus, as compared to Table 1. Furthermore, an evolutionary analysis of those 11 *Trichoderma* isolates by the maximum likelihood method was performed and presented in Figure 3. They may be divided into three clusters, with cluster A consisting of such isolates as G63/18, G109/18, G69/18, G64/18, G78/18, G379/18, G67/18, G398/18, cluster B includes the following isolates: G70/18, G65/18, and cluster C only consists of isolate G61/18. Moreover, it is worth mentioning that the fungal isolates grouped in cluster A were the most active, mainly with an antagonistic activity against three or more pathogens. The fungi of cluster B had the weakest antagonistic activity—only against one pathogen each, *Colletotrichum* spp. and *Verticillium* spp., respectively, while the only isolate present in cluster C was active against two pathogenic fungal genera *Botrytis* and *Verticillium*.

To better describe the diversity in terms of the range of antagonistic interactions between the 11 chosen *Trichoderma* isolates against phytopathogenic fungi resulting from *Botrytis* spp., *Colletotrichum* spp., *Verticillium* spp., *Phytophthora* spp. implied diameters of growth and/or sporulation inhibition were measured in another experiment on Petri dishes. The results are presented in Table 2.

As for *Trichoderma* spp. interaction with *Botrytis* spp. (G275/18, G277/18, G276/18) the diameter of the *Botrytis* sp. inhibition zone ranged from 14.5 mm when *Trichoderma* sp. isolate G67/18 interacted with *Botrytis* sp. G276/18, to 71.5 mm in the interaction of *Trichoderma* sp. G64/18 against *Botrytis* sp. G276/18. All of the tested *Trichoderma* isolates revealed a variable growth inhibition. G379/18 and G398/18 did not cause the growth inhibition of *Botrytis* sp. G276/18.

However, those two isolates showed unequivocal sporulation inhibition zones of these *Botrytis* sp. isolates (44.5 and 47.4 mm, respectively). Similarly, *Trichoderma* sp. G63/18, G65/18, G67/18, G69/18 in relation to *Botrytis* sp. G276/18 and G67/18 in relation to G277/18 revealed not only growth inhibition but also additional sporulation inhibition.

When describing the relationship of *Trichoderma* spp. towards *Colletotrichum* spp. (G172/18, G371/18, G166/18) the range of the diameter of the growth inhibition zone started from 15.3 mm when *Trichoderma* sp. G109/18 engaged *Colletotrichum* sp. G371/18 to 90 mm when *Trichoderma* sp. G61/18 has been combined with *Colletotrichum* sp. G166/18. Only *Trichoderma* sp. G379/18 and G398/18 inhibited *Botrytis* spp. when tested via sporulation, but not causing the inhibition of growth.

Going forward, when comparing the interactions of the tested *Trichoderma* isolates with *Verticillium* spp. (G293/18, G296/18, G297/18) simultaneous growth inhibition was observed far more often than sporulation inhibition. Out of the 11 *Trichoderma* isolates tested, only four isolates did not show sporulation inhibition against *Verticillium* sp. G293/18, three against G296/18, and four against G297/18 but rather produced a reasonable decrease of its growth. The growth inhibition diameter zone ranged from 20.7 mm for *Verticillium* sp. G293/18 as influenced by *Trichoderma* sp. G78/18 to 63.1 mm for *Verticillium* sp. G297/18 as influenced by *Trichoderma* sp. G379/18. The isolate *Trichoderma* sp. G379/18 did not inhibit the growth of *Verticillium* sp. G296/18, while *Trichoderma* sp. G65/18 and G398/18 did not inhibit the growth of *Verticillium* sp. G297/18.

Similar to *Colletotrichum* spp., *Phytophthora* spp. (G368/18, G373/18, G369/18) growth was not inhibited by *Trichoderma* isolates G379/18 and G398/18, and G368/18 was not inhibited by G61/18. However, the aforementioned *Trichoderma* isolates revealed sporulation inhibition, except for G398/18 with *Phytophthora* sp. G368/18. The lowest growth inhibition zone diameter was noted when *Trichoderma* sp. G65/18 interacted with *Phytophthora* sp. G368/18 (12.7 mm) and the largest when *Trichoderma* sp. G70/18 interacted with *Phytophthora* sp. G368/18 (90 mm). The number of isolates that inhibited the growth of *Phytophthora* spp. was greater than for *Botrytis* spp. and *Colletotrichum* spp., but smaller than for *Verticillium* spp.

#### 2.1.2. Enzymatic Activity of Selected *Trichoderma* Isolates

The results of the enzymatic activity of selected *Trichoderma* isolates such as cellulases (FPU), carboxymethylcellulases (CMC), *β*-glucosidase (BGL), xylanase (XYL), protease (PRO), amylase (AMY) are presented in Table 3.

Among the 11 tested isolates of *Trichoderma* spp., nine showed FPU activity at pH 7 and at a temperature of 37 °C, five at pH 4.5 and 37 °C, eight at pH 7 and 50 °C and eight at pH 4.5 and a temperature of 50 °C. They were different strains depending on the assay conditions. The highest FPU at 37 °C, pH 7 was noted for isolate G109/18 (1 μmol min^−1^ mL^−1^), at 37 °C, pH 4.5 for G398/18 (0.99 μmol min^−1^ mL^−1^), at 50 °C, pH 7 for G64/18 (1.23 μmol min^−1^ mL^−1^), at 50 °C, pH 4.5 again for G398/18 (2.02 μmol min^−1^ mL^−1^).

As for CMC activity at 37 °C and pH 7 conditions, nine *Trichoderma* spp. isolates were noted to show this activity, at 37 °C and pH 4.5 it was four isolates, at 50°C and pH 7 it was also four isolates, under the following conditions of 50 °C and pH 4.5 there were five isolates with the aforementioned activity. The highest CMC at 37 °C, pH 7 was noted for isolate G69/18 (1.15 μmol min^−1^ mL^−1^), at 37 °C, pH 4.5 for G61/18 (0.15 μmol min^−1^ mL^−1^), at 50 °C, pH 7 for G63/18 (0.77 μmol min^−1^ mL^−1^), and at 50 °C, pH 4.5 again for G64/18 (0.53 μmol min^−1^ mL^−1^).

Concerning BGL, the highest level of activity was revealed for G70/18 (254 μmol min^−1^ mL^−1^), nevertheless, all of the 11 tested *Trichoderma* isolates showed *β*-glucosidase potential.

When XYL is considered, 10 of the tested *Trichoderma* spp. showed this activity. Within this group, G65/18, G70/18, and G109/18 stood out substantially showing the following activity 2750, 1294, 1708, 1367 μmol min^−1^ mL^−1^, respectively.

In total, 8 of 11 tested *Trichoderma* isolates revealed PRO activity. The most prominent strains in this regard were isolates G67/18 and G69/18 which showed the following activity of 0.37 and 0.36 μmol min^−1^ mL^−1^, respectively.

Regarding AMY activity, 9 of the 11 tested *Trichoderma* isolates were the most prominent amylase producers. Isolate G70/18 stood out the most from the rest demonstrating 1130 μmol min^−1^ mL^−1^.

Such a wide range of hydrolytic properties allows for the possibility that the use of selected *Trichoderma* isolates in a biopreparation for raspberry naturalization can have a positive effect in terms of the efficient decomposition of organic matter if such a biopreparation were to be applied in the targeted soil environment.

#### 2.1.3. Effect of Supplement Addition on *Trichoderma* Isolate Growth

**Figure 4:**
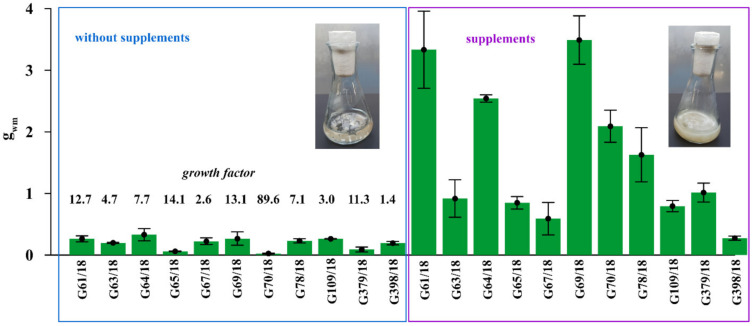
The stimulation of *Trichoderma* isolates on biomass production by 1% prebiotic supplement mixture addition (adenosine, adonitol, arabitol, erythritol, mannitol, sorbitol) in modified Mandels and Andreotti liquid medium. The growth factor calculated for each isolate showing the multiple of the increase in biomass after supplement addition. Error bars indicate a standard error, *n* = 3. The two flasks shown with the liquid culture of fungus illustrate an example of different mycelium growth following culturing with and without supplement addition.

The substantial stimulation of all 11 tested *Trichoderma* isolates on biomass production by 1% prebiotic supplement mixture addition (adenosine, adonitol, arabitol, erythritol, mannitol, sorbitol) was noted in modified Mandels and Andreotti liquid medium (Figure 4).

The intensity of this positive effect differed depending on the particular isolate. Supplement addition increased the growth of G61/18 by 12.7 times, G63/18 by 4.7 times, G64/18 by 7.7 times, G65/18 by 14.1 times, G67/18 2.6 by times, G69/18 13.1 by times, G109/18 by 3 times, G379/18 by 11.3 times and G398/18 by 1.4 times. The results indirectly, but with a high degree of probability indicate that the growth of selected *Trichoderma* isolates will be stimulated in the targeted soil environment by those supplements if applied as a biopreparation ingredient.

### 2.2. The Early Effect of the Trichoderma Isolates on Raspberry and Soil Properties

**Figure 5:**
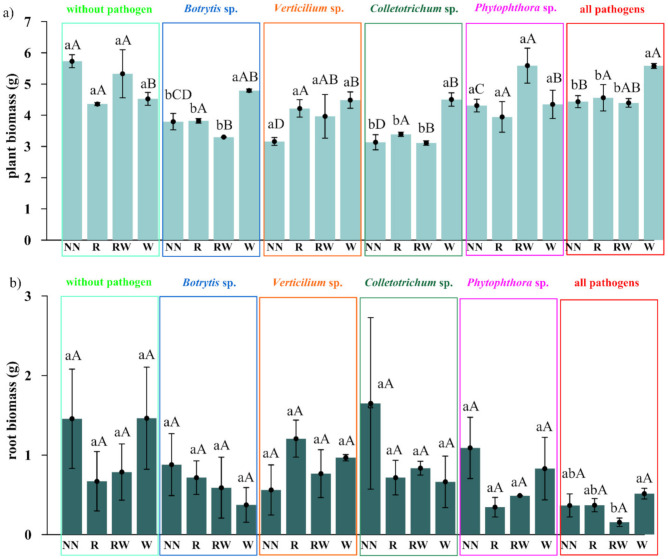
The early effect of the *Trichoderma* isolates on *Polana* raspberry plant growth in the pot, the experimental results depended on the pathosystem and naturalization strategy applied. (**a**) Dry weight of the biomass of the above-ground plant material (leaves and stem), (**b**) fresh weight of the biomass of the roots, according to pathosystems *Botrytis*, *Verticillium*, *Colletotrichum*, *Phytophthora*, and all pathogens (*Botrytis*–*Verticillium*–*Colletotrichum*–*Phytophthora*) and without pathogens, within different *Trichoderma*-naturalization strategies: no naturalization (NN), root inoculations (R), root inoculations and watering (RW), watering (W). Error bars indicate a standard error, *n* = 3, different small letters above the bars indicate homogeneous groups within the particular pathosystem, different capital letters above the bars indicate homogeneous groups within the particular naturalization approach (according to ANOVA with *p* < 0.05).

**Figure 6:**
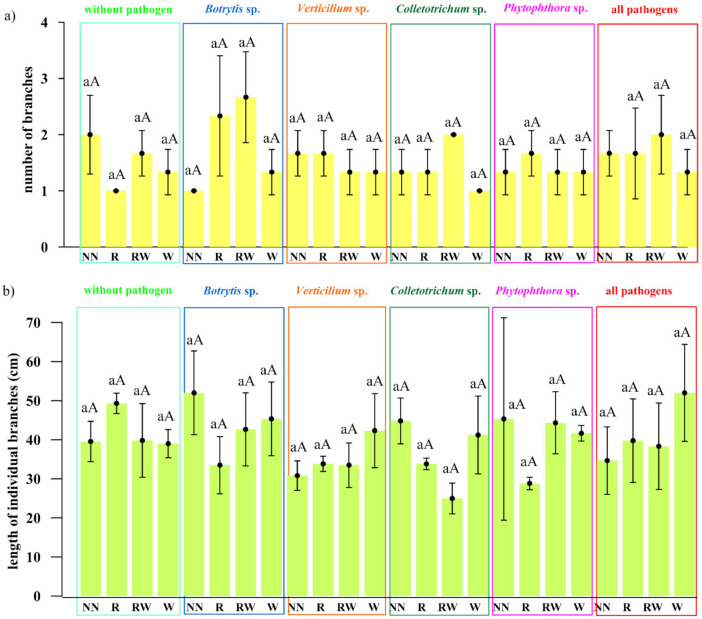
The early effect of the *Trichoderma* isolates on *Polana* raspberry plant growth in the pot experiment depended on the pathosystem and naturalization strategy applied. (**a**) The number of branches, (**b**) the length of individual branches (cm), according to pathosystems *Botrytis*, *Verticillium*, *Colletotrichum*, *Phytophthora*, and all pathogens (*Botrytis*–*Verticillium*–*Colletotrichum*–*Phytophthora*) and without pathogens, within different *Trichoderma*-naturalization strategies: no naturalization (NN), root inoculations (R), root inoculations and watering (RW), watering (W). Error bars indicate a standard error, *n* = 3, different small letters above the bars indicate homogeneous groups within the particular pathosystem, different capital letters above the bars indicate homogeneous groups within the particular naturalization approach (according to ANOVA with *p* < 0.05).

The effect of *Trichoderma* isolates on *Polana* raspberry plant growth in a pot experiment within a pathosystem (*Botrytis*, *Verticillium*, *Colletotrichum*, *Phytophthora*, all pathogens, without pathogens) and with an applied naturalization strategy (no naturalization (NN), root inoculations (R), root inoculations and watering (RW), watering (W)) were tested. The results of the above-ground plant material dry weight of biomass (leaves and stem) are presented in Figure 5a and the fresh weight of biomass of the roots in Figure 5b, the number of branches in Figure 6a and the length of individual branches in Figure 6b. A very early raspberry response to *Trichoderma* activity was the most clearly noted difference in the above-ground biomass comparison. No early effect was noted as far as the number of branches and the length of individual branches were concerned.

The pathogen load effect was notable since significantly lower raspberry above-ground biomass was the result produced by the application of pathogen contamination as compared to an absence of naturalization treatments among the pathosystems (Figure 5a). This surely indicates that the experimental design works properly. For roots, this effect was the most pronounced within all pathogens’ contamination subjects (*Botrytis*, *Verticillium*, *Colletotrichum*, *Phytophthora*) (Figure 5a). Nevertheless, the positive, preservative effect of the *Trichoderma*-naturalization strategy was observed. Therefore, when comparing the same *Trichoderma*-naturalization strategy within different pathosystems, at least the same level of biomass is observed, this indicates its early positive, preservative effect. This effect was revealed for above-ground plant biomass in R and W naturalization strategies. The RW strategy has a positive influence on *Verticillium* sp., P*hytophthora* sp. and all pathogen pathosystems. In turn, an adverse effect was noted for the *Botrytis* sp. and *Colletotrichum* sp. pathosystems.

The significantly positive effect on biomass was noted when watering (W) was applied in *Botrytis* sp., *Colletotrichum* sp. pathosystems, and all pathogens (*Botrytis*, *Verticillium*, *Colletotrichum*, *Phytophthora*) when compared to other strategies within a particular pathosystem. This may be regarded as a very early raspberry response to *Trichoderma* activity. A trend can also be seen in the *Verticillium* sp. pathosystem in pots where *Trichoderma* was applied (R, RW, W) more biomass was found than in pots without *Trichoderma* application. For the *Phytophthora-*pathosystem it seems that the RW *Trichoderma-*naturalization strategy may also reveal the early protective effect which results in slightly improved biomass production.

No significant differences were noted in root biomass production within different protection strategies introduced in each of the examined pathosystems. However, a positive tendency in the mitigation of the negative influence of *Verticillium* sp. and all applied pathogens together, after *Trichoderma* application was noted for root inoculation (R) and watering (W), respectively. In summary, the effect of the naturalization strategy was correlated with the occurring pathogen type.

The early effect of the *Trichoderma* isolates on the nutrient contents in the plant (leaves and stem) and soil chemical properties in the *Polana* raspberry pot experiment which depended on the pathosystem and naturalization strategy applied is shown in Table 4. The macronutrients content in the stems and leaves (N, P, K, Ca, Mg), absorbable forms of minerals in the soil (P_2_O_5_, K_2_O, Mg), and also nitrogen forms in the soil (N-NO_3_, N-NH_4_, N_min_) were analyzed.

Chemical properties were compared between different *Trichoderma*-naturalization strategies within each pathosystem. No differences were noted as far as the Ca and Mg content in stems and leaves and Mg content in the soil. *Trichoderma* addition had the greatest influence on the N content in the stem and leaves.

When *Trichoderma* isolates were applied to the roots while planting (R) in the soil without pathogens contamination there was a slightly greater value of N content noted comparing to the no naturalization soil (NN). A similar situation was detected in the *Colletotrichum* sp. contaminated soil. Thus, the root (R) and root following watering (RW) naturalization strategy worked for greater N content than in the NN. The RW strategy in *Botrytis* sp., *Verticillium* sp., and all pathogen contaminations treatments tended at least towards N content maintenance at the same level as the NN strategy. Such a protective effect was not noted when *Phytophthora* sp. was present.

A relatively clear positive response was noted for the P content in stems and leaves for *Botrytis* sp. contamination when RW was applied and in all pathogen pathosystems in the R and RW strategies.

As for the K content in stems and leaves, the positive response of *Trichoderma* spp. was noted in the soil without pathogen contamination when the R strategy was applied, while for the pathosystem *Botrytis* sp. it was the RW strategy, and for *Colletotrichum* sp. the R and RW treatments produced a positive response. For *Phytophthora* sp., contamination of the W strategy at least did not lead to a lowering K content compared to NN.

In considering the P_2_O_5_ and K_2_O contents in the soil, the R strategy resulted in higher values compared to NN for such treatments in *Phytophthora* sp. and all pathogen contamination. Additionally, the K_2_O content increased when the W strategy was applied in the treatment without pathogens.

The N-NO_3_ and N_min_ soil content was observed to increase when the W strategy was applied in the treatment without pathogens, and also when the R strategy was introduced for *Botrytis* sp. when the R and the RW strategies were applied for *Colletotrichum* sp., and when the R and RW strategies were used for *Phytophthora* sp. contamination.

Finally, the N-NH_4_ content increased when the R and the RW *Trichoderma*-naturalization strategy was adjusted, but only for all pathogen variants.

### 2.3. Laboratory-Scale Biopreparation Development

#### 2.3.1. *Trichoderma* Sporulation Optimization

Having selected and characterized the antagonistic fungal isolates the next step was to provide the required and reasonable number of spores. Therefore, the sporulation intensity of the *Trichoderma* isolates on different agar, solid-state, and liquid microbiological media were compiled in Table 5. The varied reaction of the *Trichoderma* isolates to the tested media was noted.

For plates with excellent sporulation with respect to each isolate (marked as dark green) the average spore number reached 10^9^ for isolate G109/18, and 10^10^ spores for the following: G61/18, G63/18, G64/18, G65/18, G67/18, G69/18, G70/18, G78/18. No spores were demonstrated for G379/18 and G398/18.

Isolate G61/18 showed the highest preference for the PDA and WBAC-A medium and showed an inherently high level of sporulation. Isolated G63/18 sporulated very well on MSCL-A, MA, WBAP-B, WBAP-C, WBAP-D, WBAP-G. *Trichoderma* sp. isolate G64/18 revealed sporulation on MSCL-A, TR, MA, WBAP-A, WBAP-E, WBAP-H, MSCL-L, MSCL, whereas WBAP-G was the most useful medium for *Trichoderma* G65/18. Isolate G67/18 sporulated very well on WBAP-H, MSCL-L, and PDA. G70/18 was very versatile and demonstrated sporulation when culturing on MSCL-A, TR, MA, WBAP-A, WBAP-B, WBAP-C, WBAP-D, WBAP-E, WBAP-G, WBAP-H, PDA. Isolate G78/18 sporulated on MSCL-A and WBAP-F. Medium WBAP-F was also the most appropriate choice for isolate G109/18. *Trichoderma* sp. G379/18 and G398/18 were the least versatile and only produced mycelium growth on MSCL-A, WBAP-A, WBAP-B, WBAP-C, WBAP-F, WBAP-G, WBAP-H, MSCL-L.

#### 2.3.2. Formulations

**Figure 7:**
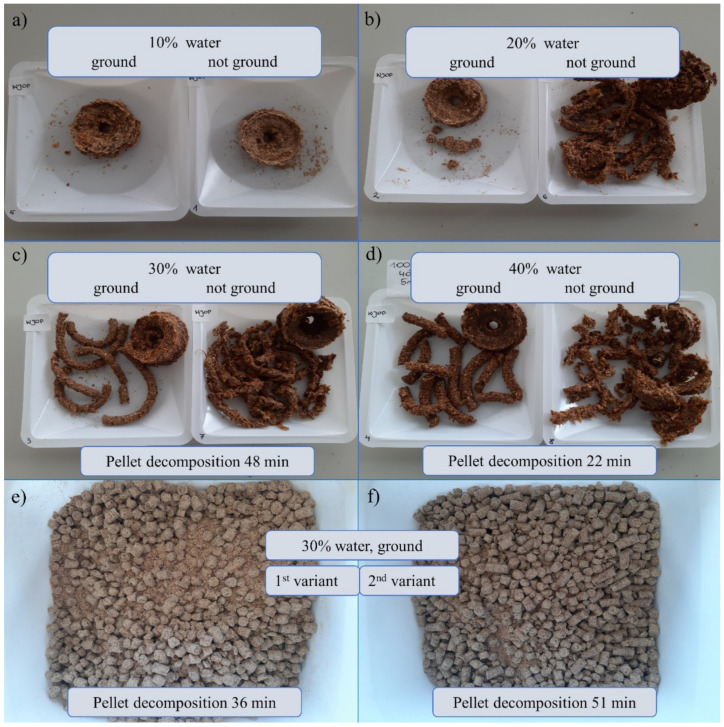
The pellet formulations depended on the amount of water addition and the presence or absence of grinding before pelleting: (**a**–**d**) prepared using a hand mincer, (**e**,**f**) by Nawrocki Pelleting Technology, (**e**) 1st variant after one passage and 2nd variant after two passages. The pellets were prepared based on wheat bran, dried and grounded apple pomace, whey protein concentrate with prebiotic supplement addition (adenosine, adonitol, arabitol, erythritol, mannitol, sorbitol), and rapeseed oil.

Pellet formulation was based on solid powder including wheat bran, dried apple pomace, whey protein concentrate with prebiotic supplement addition (adenosine, adonitol, arabitol, erythritol, mannitol, sorbitol), and rapeseed oil were prepared both on the ground and not ground material, with different amounts of water added to select an option that was compacted enough to prevent it from disintegrating immediately, but it also had a reasonably short decomposition time so that its organic ingredients become available and the microorganisms contained within it could function, multiply and exhibit antagonistic properties. The results are shown in Figure 7. The most compact pellet structures were obtained using a hand mincer and had a 30% and 40% water content, where the material was ground prior to pelleting. For these two formulations, pellet decomposition was performed. The results were 48 and 22 min, respectively. The other structures were not stable enough or perhaps even the pelleting stage failed.

The starting material for Nawrocki Pelleting Technology showed the best pelleting properties. This material was characterized by a low degree of dustiness and the best ability to be combined with oil. Both variants (after one and two passages) allowed for the attainment of a stable and homogeneous pellet without any problems with matrix clogging or the excessive caramelization of the material. It was characterized by a favorable humidity and by far the best brittleness (its hardness and brittleness were not excessive). The 1st variant of the pellet took 36 min to decompose which is more reasonable compared to the pellet prepared using the two-passage method (51 min).

Low sugar maltodextrin was proposed as a dissolvable powder formulation and this was followed by screening for different gelling agents (hydrocolloids) and water addition with the viscosity and mass of the adhering suspension tested (Table 6). Certain observations were made, for example, that the viscosity values of 150–300 mPa made the material sticky enough to adhere properly to raspberry roots, for a viscosity >300 mPa the suspension was very thick and it was also delaminating, therefore, it was hardly possible for the root material to immerse in it.

For most of the tested options, the viscosity values were very low, so this explicitly excludes the possibility of their future field application. The most likely option was 4% xanthan gum in 20% water solution, 6% xanthan gum in 15% water solution, 6% carboxymethylcellulose or guar gum in 20% water solution, or apple pectin in both tested options (15% in 20% water solution and 20% in 15% water solution).

For xanthan gum, 7.9 and 7.6 g of the suspension adhered to the raspberry roots, respectively. For carboxymethylcellulose, it was 2.68 g. In turn for guar gum, it was 7.23 g. Regarding apple pectin, it was 3.9 and 2.98 g respectively.

## 3. Discussion

### 3.1. Fungal Consortium Selection for Biopreparation

The overriding goal of the presented work was to compose a fungal consortium for the biostimulation of plant growth and resistance, including the effective control of pathogens belonging to the following genus and species *Botrytis* (including *B. cinerea*), *Verticillium*, *Colletotrichum* (including *C. acutatum*) and *Phytophthora* for the biopreparation of raspberry agroecological cultivation. It was emphasized previously that strains of *Trichoderma* spp. differ greatly in their effects on plants. This study was motivated by the fact that harnessing beneficial microbes presents a promising strategy to optimize plant growth, agricultural sustainability and smart farming [28]. An effort was made to search for prominent representatives among wild raspberry roots and rhizosphere microbiota. This approach was dictated by the current research which shows that strains of *Trichoderma* spp. differ greatly in their effects on plants [29], the locally isolated microorganisms might be more effective against local pathogens than bacteria from different regions of the world [23]. Thus, candidates from the relevant niche should be preferred [30].

Very recently [21], the challenge was undertaken to isolate from wild raspberry, in turn, bacterial representatives were chosen and characterized in terms of biocontrol ability against *Botrytis* sp., *Verticillium* sp. *Colletotrichum* sp. and *Phytophthora* sp. phytopathogens to select the best antagonists for biopreparation development. As efficacy testing in bioassays under field conditions requires significant resources and time, the antagonistic potential of candidates has often been tested in a first screening round under the in vitro conditions of agar plates allowing for rapid throughput with clearly discriminating results [31].

This very first step tending towards biopreparation construction is to ensure that the preferred and selected isolates not only reveal great potential against pathogens but also that they do not interact negatively with each other. This step is designed to ensure the efficiency of their multiplication in the environment at the further stage of the experiment. A very inadvisable scenario would be the case of beneficial microorganisms competing with each other. This is an important consideration, however, it tends to be omitted in the stepwise screenings of microorganisms for commercial use in biological control [30]. The completed research (Figure 2) indicates that in a pool of putative beneficial and antagonistic isolates there is a wide range of mutually antagonistic isolates. Nevertheless, the final selection was focused on choosing isolates that do not antagonize each other and are simultaneously not recognized as potential pathogens and also those isolates with an uncertain identification are omitted.

Thus, *Absidia* sp., *Alternaria* sp., *Cladosporium* sp., *Coniothyrium* sp., *Cryptococcus* sp., *Fusarium* sp., *Mucor* sp., *Penicillium* sp. representatives were excluded since they proved to be phytopathogens [32], as well as *Massarina* sp. representatives [33], *Pyrenochaeta* sp. [34], *Geotrichum* sp. [35], *Mortierella* sp. [36], *Plectosphaerella* sp. [37]. The genus *Umbelopsis* representatives were previously linked to the decomposition of the surface of the litter layer of forest environments, not known to have antagonistic activity [38], so they were also excluded. In turn, there were some isolates considered in the literature as the potential choice for further consortium accomplishment. These were *Apiotrichum* sp. [39], *Aureobasidium* sp., *Humicola* sp., *Podospora* sp., *Talaromyces* sp., and finally *Trichoderma* sp. [32]. The action taken resulted in the selection of the 11 *Trichoderma* isolates (Table 1) among the 159 tested (Figure 1, Table S1) where almost all of them show solid antagonistic properties against phytopathogens belonging to the following genera: *Botrytis* (including *B. cinerea* species), *Verticillium*, *Colletotrichum* (including *C. acutatum* species) and *Phytophthora*, displaying growth and/or sporulation inhibition of pathogenic representatives and also there is no negative mutual interaction. These *Trichoderma* isolates were used as biopreparations in raspberry naturalization as described and claimed in patent application P.434148 [40].

The antagonistic properties of *Trichoderma* isolates against fungal and fungal-like plant pathogens have been described previously and followed by many efforts made to utilize these organisms in plant growth, resistance stimulation [15] and biological plant protection [41,42,43]. In the literature, quite often attention is paid to the phenomena that *Trichoderma* spp. activities use lytic enzymes [14,41,44].

Enzymatic abilities may be the key to accurately understanding the achievable functions of selected isolates in the targeted environment. The broader the spectrum of secreted plant cell-wall degrading enzymes, the more environmental niches fungi can develop in, and the more competitive they can be against existent pathogenic microorganisms [45,46]. A reasonably wide range of hydrolytic properties demonstrated for the chosen *Trichoderma* spp. is proof that the use of selected isolates in a biopreparation for raspberry naturalization can have a positive effect in terms of the efficient decomposition of organic matter. Hydrolytic enzymes that degrade cellulose facilitate the colonization of *Trichoderma* into the tissue [47]. The cellulolytic abilities presented by all of the examined isolates may contribute to an increase in the number of ingredients available to the plant [48]. It is also worth noting that the addition to the soil of microorganisms that have a high degree of enzymatic activity may stimulate different processes in the soil environment [49] and drive the soil ecosystem services that are important in soil quality evaluation [50].

### 3.2. Evaluation of the Influence of the Promptly Selected Trichoderma-Based Consortium on Raspberry in the Pot Experiment

Having selected the efficient *Trichoderma* isolates, we seek to achieve a positive influence on raspberry plants and soil-associated properties. Thus, a raspberry pot experiment was set up which coincides with the *Botrytis cinerea*, *Verticillium* sp., *Colletotrichum acutatum*, *Phytophthora* sp. pathosystems, and different *Trichoderma*-naturalization strategies, such as root inoculations and watering. We considered the differential reaction in raspberry biometrics, as well as the diverse responses of selected plant macro- and micronutrient contents, depending on the pathosystem and naturalization strategy applied.

The pathogen load effect was notable since lower raspberry biomass was determined in soils where pathogens contamination was applied. Pathogens weakened biomass production when compared to the control. This effect was more clearly shown in the aboveground biomass comparison (Figure 5a), than in the roots (Figure 5b). For the roots, this effect was most pronounced in the treatment where all of the tested pathogens were introduced. Nevertheless, the sought-after, positive, preservative effect of the *Trichoderma*-naturalization strategy was also observed.

A significant difference was encountered when water (W) was applied to *Botrytis cinerea*, *Colletotrichum acutatum* and the pathosystems of all pathogens (*Botrytis cinerea, Verticillium* sp., *Colletotrichum acutatum, Phytophthora* sp.), while comparing to other strategies for aboveground biomass production. A trend may be observed in the *Verticillium* sp. pathosystem which was present in the pots where the *Trichoderma* consortium was applied (R, RW, W) a greater amount of biomass was found than in the pots without *Trichoderma* application. For the *Phytophthora*-pathosystem, it seems likely that the RW *Trichoderma*-naturalization strategy may also reveal a protective effect conveying slightly improved biomass production. This may be regarded as a very early raspberry response to *Trichoderma* activity due to the short period of its possible impact (two months of the experiment). No significant differences were found in root biomass production within the different naturalization strategies introduced in each of the examined pathosystems. However, a positive tendency in increasing fresh weight of roots biomass was noted after root inoculation in *Verticullim* sp. pathosystem and after *Trichoderma* spp. watering when all pathogens were applied.

A certain degree of correspondence was noted between the effectiveness of the naturalization strategy in terms of the above-ground biomass increase with the type of pathogen applied. For *Botrytis cinerea*, *Colletotrichum acutatum* and *Verticillium* sp., this would seem to be overly optimistic given the fact that once applied *Trichoderma* consortium could be effective. In order to affect the plant response to *Phytophthora* sp. it would seem that only one application is insufficient, but the reapplication strategy seems promising.

Noted differences which occur as a result of the application of the *Trichoderma* consortium may suggest that *Trichoderma* isolates were able to colonize the root system and to persist for the entire lifespan of this crop. The detailed experiments required to prove this were performed previously on the model of e.g., maize plants [51]. Fungal colonization most likely stimulated plant growth through factors that aid in nutrient uptake, auxin, and siderophore production as described e.g., by Eslahi et al. and Jaroszuk-Ściseł et al. [15,52]. A symbiotic relationship may be established in which *Trichoderma* improves plant growth and development through increasing the systemic resistance of the plant against possible attacks from pathogens, thereby increasing tolerance to any stresses and improving the capacity of active plant growth stimulation [53].

We are aware that plant biomass production changes do not reflect the entire and indeed, the possible *Trichoderma* sp.–pathogen–raspberry relationship, especially since various factors influence this bond. Thus, future, long-term experiments are required, to determine additional parameters and outcomes including e.g., fruit yield evaluation [54], soil enzymatic activity [55], a summary of the presence of pathogens [24], the plant growth-promoting microbiome network [56], or simply the symptoms of diseases caused by those pathogens. Nevertheless, the obtained results cumulatively produce a positive *Trichoderma*-based consortium response and may therefore encourage further research in this area.

### 3.3. The Development of the Biopreparation Targeted at Raspberry Naturalization

The step-by-step development of the biopreparation targeted at raspberry naturalization was described. This proposal included an assortment of *Trichoderma* spp. culture medium compositions, carriers, and finally prebiotic supplement addition. The primary motivation was to produce tolerably low-cost formulation concepts to develop different practical application methods of the *Trichoderma*-based consortium. Firstly, the ready-to-use formulations were to be introduced during the planting period (pellets, gel). Secondly, the variant was to be applied as naturalizing watering or spraying (soluble powder). These formulations are widely recognized to be useful in agricultural applications [57,58].

Since there is a wide range of organic waste that should be utilized and the economically significant role of microorganisms in the bioconversion of solid wastes has been documented [59], the current trend towards organic waste management in agriculture is being recognized [60]. Therefore, we decided to propose among other factors pellet formulation including such carriers as dried apple pomace and wheat bran.

The proposed carrier assortment was dictated by previous prominent findings [17]. Thus, apple pomace application for agricultural purposes may be especially important for local apple processing companies surrounded by orchards and crops, which often lack sources of exogenous organic matter [17]. The agricultural use of fruit pomace as a natural fertilizer promoting plant growth in organic farming [61,62] and also as a source of phytochemicals that act as natural bio-fungicides has been studied previously [63]. Pomaces have already proven useful for promoting the growth of plants and facilitating the achievement of higher yields, due to the organic matter and nutrients introduced. It should also be emphasized that previous research proved that additional fungal species may be incorporated easily into the soil with apple pomace as a carrier [17].

An efficient method is the following, the microorganism is cultured on different types of organic matter, including agricultural wastes and by-products which are available at low cost [64]. Additionally, wheat bran-based technologies for beneficial microorganism inocula used as biofertilizers were previously tested [65,66]. Furthermore, since amino acids serve as nitrogen sources for microorganisms [67] we combined apple pomace and wheat bran with whey protein concentrate addition. We assumed that the long list of amino acids in the proposed protein concentrate included tyrosine, phenylalanine, histidine, glycine, arginine, valine, serine, proline, isoleucine, alanine, threonine, lysine, leucine, glutamic acid and aspartic acid which should fulfil fungal requirements.

The kind of carrier utilized defines the physical form of the biofertilizer [65]. The activity of mixing the abovementioned ingredients only leads to an insoluble solid powder formulation, which is rather difficult to spread in field conditions and as a consequence, raspberry producers are not eager to use this. It was demonstrated that 5% rapeseed oil and 30% water additions serve to make the biomass adequate for pelleting with a reasonable period of pellet decomposition (Figure 7).

Significant progress has been made in recent years with regard to developing the formulations of plant beneficial microorganisms through entrapment in natural water-soluble polymer-based carriers and their application as biostimulants [68]. The authors have summarized that there is a wide selection of additional materials used in bioimmobilized systems, which may serve as carrier bulking agents, enhance formulation stability, materials that protect and feed microbial cells or spores such as clay minerals, skimmed milk, chitin and chitosan, starch, humic acids, protein hydrolysates and finally sugars. In recent times several authors have claimed that maltodextrin is a good excipient for microbe-based products [69,70]. Maltodextrin is easily dissolvable and digestible, being absorbed as rapidly as glucose [71]. It may serve as a dry powder formulation element and a good carrier for microorganisms [72] that can be dispersed in a water solution before plant watering (irrigation) or spraying. It has been proven in this research that maltodextrin along with xanthan gum, carboxymethylcellulose, guar gum or apple pectin with adequate water addition (Table 6) may be efficient with regard to *Trichoderma* spp. gel-entrapment, thereby unequivocally demonstrating viscosity at a level that is sufficient to stick to the raspberry root in such a gel form. To the best of our knowledge, no previous research has been performed with which to assemble pellets including coincident apple pomace, wheat bran, and whey protein.

Maltodextrin was in turn previously used as the wall material in spray-drying encapsulation [73], but not in entrapment formulation influencing the gelling process. An additional and quite novel element of the biopreparation assembly concept was prebiotic supplementation. We latterly presented a case study report concerning nutritional competition between *Trichoderma* spp. isolated from wild raspberries and fungal phytopathogenic isolates such as *Colletotrichum* sp., *Botrytis* sp., *Verticillium* sp. and *Phytophthora* sp. (the same as those utilized in the presented article) [4]. The extent and nature of the competition was evaluated based on nutritional potentiates. Namely, these were consumption and growth, which were calculated based on substrate utilization located on Biolog^®^ (Hayward, Canada) Filamentous Fungi (FF) plates. The niche size, total niche overlap and *Trichoderma* competitiveness indices along with the occurrence of a stressful metabolic situation with regard to substrates highlighted the unfolding approach. Therefore, the *Trichoderma* spp. and pathogen niche characteristics were provided.

As a result, the substrates in the presence of which *Trichoderma* spp. nutritionally outcompeted the pathogens were identified. These were adonitol, arabitol, erythritol, glycerol, mannitol, and sorbitol. This preliminary research determined that selected substrates may serve as additives in biopreparations of *Trichoderma* spp. dedicated to plantations contaminated by phytopathogens. The previously performed tests were in a plate microscale (where the amount added is not known due to producer confidentiality), but in the present evaluations, the positive effect was confirmed in a flask-scale growth (Figure 4). Supplement addition improved the biomass production of *Trichoderma* spp. It was assumed in advance that an intentionally small amount of additives should be tested to minimize biopreparation production costs by reducing the high cost of particular supplements. Therefore, we focused on the application of 1% prebiotics content. Research concerning prebiotic supplement addition to biopreparations requires additional experiments as well as costing and profitability calculations. Thus, raspberry pot and field experiments within a variable pathosystem are planned, in which not only fungal consortium applications are evaluated but there is also a search for possible differences where the complex biopreparation (microorganisms and other ingredients) is applied. It is important to emphasize that different carriers have various structures and diverse fungi can grow on them, thus they can influence the stability of the biofertilizer [74], therefore carriers should be selected separately for each type of microorganisms used in biopreparations. Moreover, plain carriers alone may influence the inherent microbial consortium of the soil [75]. The effectiveness of prebiotic supplementation to biopreparations should then be an asset through in vivo investigation.

Last but not least, an important aspect of biopreparation assembly is spore production. It is suggested to perform preliminary tests on mass production using readily available, simple, and inexpensive agar media with a Petri plate assay and to discard isolates producing less than 1 × 10^5^ spores per plate on a common agar medium [30]. In accordance with this plan, a stepwise assessment of the biomass production of candidate antagonists in rapid-throughput screening agar medium systems was performed. The following agar media were examined: Potato Dextrose Agar (PDA), Malt Extract Lab Agar (MEA), Corn Meal with glycerol (CM), modified agar Mandels and Andreotti with (MA), soy flour, cellulose, lactose agar medium (MSCL-A), yeast and malt extract minimal agar medium (TR) and minimal agar medium with cellulose (MN). In total, 9 out of the 11 tested *Trichoderma* isolates fully met these quantitative requirements after thorough media screening (Table S2) to optimize its production (Table 5). In fact, the isolates differed in spore production depending on the medium used which indicates that this is a medium of specific components that regulate the sporulation bioassay.

One thing is for certain, attention should be paid to utilizing sufficient medium for particular isolates for the purposes of massive sporulation. On the other hand, the exclusion of previously patent-protected applications or commercial media at this early stage of product development will avoid unnecessarily high costs during the later steps [30]. That was the motivation of elaborating on a brand-new medium.

As basic ingredients apple pomace, wheat bran, and whey protein concentrate (WBAP) were chosen. Apple pomace and wheat bran are waste products and are also quite inexpensive and easily accessible as described above within the carrier discussion section. Several ingredients in addition to this basic formula were tested based on the literature to date, in the agar medium version. These were supplements [4] for WBAP-A, CaCl_2_ and KH_2_PO_4_ [76] for WBAP-B, with microcrystalline cellulose and soy flour for WBAP-C, microcrystalline cellulose, soy flour, CaCl_2,_ and KH_2_PO_4_ [77] for WBAP-D. For the solid-state medium version, these were grounded straw and microcrystalline cellulose [78] for WBAP-F, microcrystalline cellulose [79] for WBAP-G and sawdust [80] for WBAP-H.

In this way, isolates G64/18 and G70/18 were growing well only with the apple pomace, wheat bran, and whey protein, and thus they do not require additional ingredients in their media to sporulate well. Isolates G63/18 and G65/18 require microcrystalline cellulose addition. In turn, G69/18, G78/18, and G109/18 need a straw and microcrystalline cellulose addition. Finally, G61/18 needs some easily digestible forms of carbon (as supplements tested here: adonitol, arabitol, erythritol, glycerol, mannitol, and sorbitol).

Composing a microbial consortium for agroecology consisting of many isolates is undoubtedly an advantage due to the wide spectrum of expected diverse and positive impacts on the plant–soil system. Nevertheless, this application requires a prior individual approach towards each isolate medium elaboration. It is rather difficult to compose a universal biomass production medium for each isolate. However, the presented research shows that changing a particular ingredient may bring the formula closer to success. In this step, we operate with the awareness that further steps are required to develop a successful formula, especially concerning such key parameters as particle size, water activity, viscosity, and the concentration of solids [81].

The method used to grow *Trichoderma* to obtain spore biomass is strongly influenced by the target form of the product. For pellets, it is cost-effective to follow solid-state fermentation, where the excess substrate components will then be used as a whole to form the pellets. For watering or spraying application, no solid particles are allowed so as not to block the devices used. Thus, serious consideration should be given to the question as to whether the costs and waste utilization are reasonable for industrial-scale production—washing out spores from solid biomass or growing on an agar medium, which in the latter case is associated with reasonably straightforward spore separation.

## 4. Materials and Methods

### 4.1. Isolates Description and Characterization

#### 4.1.1. Isolation and Identification

Two fungal collections were used in this study. The first one was a collection of wild raspberry fungal isolates (159 in total). Particular isolates were obtained from wild raspberries found growing in the forests of Poland. Raspberry roots and soil were collected as described in Oszust et al. [4] from the following Forest Districts: Łuków, Świdnik, Janów Lubelski, Kraśnik, and Siedlce. The fungi were isolated from the root, rhizosphere or rhizoplane of the raspberry using serial dilution or by placing the sample material on agar media (Rose Bengal Agar with Chloramphenicol (Biocorp^®^, Warszawa, Poland), Pikovskaya Agar Medium [82], Potato Dextrose Lab Agar (A&A Biotechnology^®^, Gdańsk, Poland) or Sabouraud Dextrose Agar (Biocorp^®^, Warszawa, Poland).

The second collection was a set of phytopathogens of soft fruit plants, fungi belonging to *Colletotrichum* sp., *Botrytis* sp., *Verticillium* sp., and the fungal-like microorganism *Phytophthora* sp. Individually, these were *Colletotrichum* spp. G166/18, G172/18, G371/18, *Botrytis* spp. G277/18, G275/18, G276/18, *Verticillium* spp. G293/18, G296/18, G297/18, and *Phytophthora* spp. G408/18, G368/18, G369/18, and G373/18. More descriptive information concerning the selected strains was presented by Malarczyk et al. [83].

All of the isolates were subjected to identification on the genus/species level as previously described [21]. The sequences obtained were submitted into the GenBank (NCBI) strain database (www.ncbi.nlm.nih.gov/genbank/). An evolutionary analysis of the selected *Trichoderma* isolates was constructed using MEGA X software (University Park, Pennsylvania, USA) [84], inferred by using the maximum likelihood method and the Tamura–Nei model [85].

Details concerning the tested isolates are presented in Table 1, which gives the source and institution in which they were isolated, the GenBank accession numbers of particular sequences, GPS-coordinates of the location where the raspberry sample was obtained within the Forest District mentioned, and finally, the agar medium which was used for fungi isolation.

#### 4.1.2. Antagonistic Abilities of Raspberry Fungal Isolates against Phytopathogens

In the first step, the antagonism of raspberry fungal isolates vs. an isolate of a pathogen (*Colletotrichum* spp. G166/18, G172/18, G371/18, *Botrytis* spp. G277/18, G275/18, G276/18, *Verticillium* spp. G293/18, G296/18, G297/18, and *Phytophthora* spp. G373/18, G368/18, G369/18) was tested in pairs in Petri plates with a diameter of 90 mm on an agar medium with potato dextrose (PDA, A&A Biotechnology^®^, Gdańsk, Poland) with antibiotics addition (streptomycin and chlortetracycline). The entire surface of the medium on the plate was inoculated with 100 µL of a spore suspension of a given pathogen isolate, with a transmittance of 70% T, and a PDA medium disc with the culture of individual *Trichoderma* spp. being placed at the centre of the plate to the culture at 26 °C. Controls of all pathogenic isolates were provided without any treatment. After incubating the pathogenic fungus and the antagonist’s variant for about 5 days, culture growth and/or the inhibition zone (mm) were measured. The experiment was set up in triplicate for each pathogen (*n* = 3). The antagonism tests followed the aforementioned descriptions with minor modifications [17]. The mutual antagonistic activity between isolates has also been determined. In this step, the antagonism abilities rating was used (+++, ++, +, − for excellent, very good, good, and no response, respectively).

In the second step, the antagonism abilities of the selected *Trichoderma* isolate (G61/18, G63/18, G64/18, G65/18, G67/18, G69/18, G70/18, G78/18, G109/18, G379/18, G398/18) were determined and the results were presented as diameter zones of growth inhibition and/or sporulation mean values with a standard error being provided.

#### 4.1.3. Enzymatic Activity of Selected *Trichoderma* Isolates

The hydrolytic abilities (cellulases (FPU), carboxymethylcellulases (CMC), amylase (AMY), xylanase (XYL), *β*-glucosidase (BGL), protease (PRO)) of 11 *Trichoderma* spp. isolates (G61/18, G63/18, G64/18, G65/18, G67/18, G69/18, G70/18, G78/18, G109/18, G379/18, G398/18) were determined in the post-culture fluid in a separate experiment, after culturing *Trichoderma* spp. for 4 days in a medium composed of: lactose—5 g/L, microcrystalline cellulose-containing at least a 50% particles > 32 µm—10 g/L, soy flour—20 g/L, KH_2_PO_4_—6.3 g/L, NH_4_NO_3_—2.5 g/L, CaCl_2_ × 2H_2_O—0.82 g/L, MgSO_4_ × 7H_2_O—0.82 g/L, Tween 80—0.15%, 20 mL/L micronutrients (FeSO_4_ × 7H_2_O—513 mg/L, MnSO_4_ × H_2_O—166 mg/L, ZnSO_4_ × 7H_2_O—8.5 mg/L, CoCl_2_ × 6H_2_O—204 mg/L), Antifoam B emulsion—5 mL/L, under aerobic conditions, at pH 4.5, at 26 °C in 0.5 L flasks, 150 mL of medium, 105 rpm rotation, inoculum: 10^3^ conidial spores per flask [77]. This liquid medium is based on soy flour, cellulose, lactose (hereinafter referred to as MSCL-L) as previously described by the authors for the effective production of lytic enzymes by the *Trichoderma atroviride* G79/11 strain representative.

The hydrolytic enzymes activity was determined in liquid culture filtrates and based on cellulolytic activity (FPU) [86,87] and carboxymethylcellulases [88] after 60 min of incubation at 50 °C and 37 °C vs. 4.5 and 7.0 buffer pH, *β*-glucosidase [89], xylanase [90] amylase [91], protease [92]. These assessments were carried out with a triplicate analysis (*n* = 3) using colorimetric methods involving a spectrophotometric microplate reader Infinite M200PRO (Tecan^®^, Männedorf, Switzerland).

#### 4.1.4. Effect of Supplement Addition on *Trichoderma* Isolate Growth

The stimulation of *Trichoderma* biomass production by the mixture following supplement addition, adenosine, adonitol, arabitol, erythritol, mannitol, sorbitol (in an equal weight ratio, in total 10 g/L), was tested after 96 h in a liquid culture. The medium consisted of 0.1 M citrate buffer pH 5.6 (480 mL/L), (NH_4_)_2_SO_4_ (3 g/L), KH_2_PO_4_ (4 g/L), MgSO_4_ (0.6 g/L), CaCl_2_ × 2H_2_O (0.1 g/L), Tween 80 (0.5 g/L), a micronutrients solution (20 mL/L) (FeSO_4_ × 7H_2_O (250 mg/L), MnSO_4_ × H_2_O (80 mg/L), ZnSO_4_ × 7H_2_O (70 mg/L). The culture was kept under aerobic conditions, at pH 4.5, at 26 °C in 50 mL flasks, 15 mL of medium, 105 rpm rotation, 1 mL 10^3^ conidial spores per flask inoculum. A control was provided for each isolate culture which consisted of the medium and culture conditions described above but without the addition of supplements. The dry weight of the biomass produced was compared. The cultures were provided in triplicate (*n* = 3). This is a minimal medium [93] modified by Oszust et al. [77] (hereinafter referred to as MA-L). The main modification was that cellulose and peptone addition was omitted, but sugars were provided (glucose (10 g/L) and also saccharose (10 g/L)).

In detail, the supplements were as follows: adenosine 99% (Pol-Aura^®^, Dywity near Olsztyn, Poland), adonitol 98% (Acros Organics^®^, Waltham, Massachusetts, USA), D-arabitol (Apollo Scientific^®^, Cheshire, United Kingdom), D(-)-sorbitol (pure for microbiological analyses, pH 5–7 (100 g/L, H₂O, 20 °C)) (Merck^®^, Darmstadt, Germany), D(-)-mannitol for microbiological analyses, pH 5–7 (100 g/L, H₂O, 20 °C) (Merck^®^, Darmstadt, Germany)), i-erythritol table-top sweetener (Vivo^®^, Częstochowa, Poland). The supplements for this experiment were chosen according to previous research performed by Oszust et al. [4] using Biolog^®^ (Hayward, Canada) FF plates.

### 4.2. Effect of the Trichoderma Isolates on Raspberry Growth and Soil Properties

#### 4.2.1. Phytotron Raspberry Pot Experiment Set up—Pathosystems and Naturalization Strategies

Seedlings of the *Polana* raspberry variety were planted in pots with a diameter of 19 cm. Each pot contained 3 kg of soil (pH 5.8) obtained from a field where organic raspberry was previously cultivated and maintained in good health, without phytopathogen infection. One raspberry seedling with equally trimmed stems and roots was planted in each pot. The surface of the pots was completely covered with a mixture of vermiculite and perlite (1:1) to prevent the soil from drying out extensively. As soon as the soil was brought from the field, it was carefully mixed and then passed through a 5-mm sieve for further use in pots. The soil moisture level in the pots was kept constant at 18–20% throughout the experiment. The actual moisture level was measured using a moisture sensor which employs time-domain reflectometry (TDR) and the experimental setup was filled with an adequate amount of tap water which was fed into the stands (diameter 23 cm). The pot experiment was set up in triplicate (three pots per variant, *n* = 3) and performed in a phytotron room. The raspberry plants were grown in the phytotron room under natural sunlight supplemented by white light at a light intensity of 320 μmol m^−2^ s^−1^ using a photoperiod of day/night set to 16/8 h, with the temperature ranging from 20 to 22 °C over two months (January and February 2020).

The following six variants of treatment, including five variants of contamination with pathogens (pathosystems) were used: (a) *Botrytis cinerea* G277/18, (b) *Verticillium* sp. G296/18, (c) *Colletotrichum acutatum* G172/18, (d) *Phytophthora* sp. G408/18, (e) the variant including all of the above-mentioned tested pathogens applied together and (f) a control without the addition of pathogens, but with water addition instead of contamination.

Pathogen inoculation proceeded as follows: 10 mL of a freshly prepared sterile water suspension of the respective pathogens containing 10^8^ conidia per mL was applied to each pot in the case of a single pathogen variant. For all of the tested pathogen variants, the suspension consisted of 2.5 mL of 10^8^/mL conidia of each pathogen applied together. The pathogen inoculums were applied twice during the pot experiment. Once during raspberry planting (pipetted into the soil near the roots) and for the second time, one month after raspberry planting. In this case, the *Phytophthora* and *Verticillium* suspensions were introduced to the soil near the roots, while *Botrytis* and *Colletotrichum* were used to inoculate the plant using sterile inoculation sticks. This application method was selected due to its attacking mode [3].

For each variant of pathogen contamination, there were four naturalization variants applied, based on naturalization with the *Trichoderma* consortium as listed in the *Trichoderma* isolates section. For the first naturalization variant, the consortium was applied on the raspberry roots during planting (while setting up the experiment) (R). For the second one, the consortium was applied to the raspberry roots during planting, and after one month, water for naturalization including the consortium was applied to the soil directly to the pots (RW). The third variant was only watered with the *Trichoderma* consortium one month after raspberry planting (W). The fourth one was left without naturalization, where no *Trichoderma* spp. were applied, but only water instead (NN). Naturalization watering was applied one month after raspberry planting.

The proposed naturalization strategies were designed to reflect the possible scenarios for the plantations. Application to the roots only (R) and application of the consortium to the roots followed by watering with consortium solution (RW) are the most suitable methods to be used for newly established plantations, whilst watering only (W) was applied to already existing plantations where the treatment with microbes is possible only through watering.

Similarly, to pathogen inocula, the consortium of *Trichoderma* was applied in a 10 mL total volume of conidia suspension (0.91 mL of each *Trichoderma* isolate) with a concentration of 10^8^/mL into the soil regardless of the naturalization variant (R, RW, W).

To obtain the proper amount of fungal conidia for the pot experiment, the tested isolates were cultured on a PDA medium at 26 °C for two weeks. After that, the required concentration was provided through serial dilution, based on Thoma cell counting chamber results (Blaubrand^®^, Wertheim, Germany).

#### 4.2.2. Plant and Soil Analyses

The physicochemical analyses of the plant and soil material from the pot experiment were performed by District Chemical and Agricultural Station in Lublin according to standard methodology. Plant material (leaves and stem) testing was performed as indicated by foliar feeding (N, P, K, Ca, Mg). From the soil, there was a determination of the N-mineral content, pH, P_2_O_5_, K_2_O, Mg, and also the organic carbon and soil organic matter contents.

The determination of phosphorus (P) in the plant material after mineralization in H_2_SO_4_ and H_2_O_2_ was performed through the use of the vanadomolibdene method (KQ/PB-24). The method is based on the spectrophotometric measurement of the intensity of the yellow color of the phosphorus–vanadium–molybdic acid complex, which is formed by orthophosphate and vanadium ions in the presence of molybdate in an acidic environment with a SPEKOL-11 (Carl Zeiss Jena^®^, Oberkochen, Germany) colorimeter being used.

The determination of nitrogen content (N) in the plant material was performed by using the distillation method after mineralization in H_2_SO_4_ and H_2_O_2_. The determination of the nitrogen content consists of converting the amide form of nitrogen into ammonia through mineralization in concentrated sulfuric acid (VI), distilling ammonia from the alkaline medium, absorption in a known volume of the standard sulfuric acid solution, and titration of the excess acid with a standard sodium hydroxide solution (KQ/PB-70) using Buchi^®^ (Postfach, Switzerland) B-324 distillation apparatus with a digital burette. The determination of the magnesium (Mg) content in the vegetable material was performed after mineralization in H_2_SO_4_ and H_2_O_2_ (KQJPB-26). The principle of the method consists of measuring the absorption of radiation by magnesium atoms released when the test solution is sprayed in an acetylene-air flame using a PerkinElmer^®^ (Waltham, Massachusetts, USA) atomic absorption spectrometer.

The determination of potassium (K) and calcium (Ca) content in the vegetable material was performed after mineralization in H_2_SO_4_ and H_2_O_2_ (KQ/PB-25). The radiation emitted by a suitably excited sample was measured. The excitation source was a burner flame—propane–butane–air. The equipment used was a Jenway^®^ (Cole-Palmer, United Kingdom) flame photometer.

The determination of nitrate and ammonium content in the soil (N-NO_3_, N-NH_4_) was performed using a colorimetric method with a SKALAR^®^ (Breda, Netherlands) SCAN ++ SYSTEM flow autoanalyzer after extraction in 1% K_2_SO_4_ (KQ/PB-71). The nitrate nitrogen-reduction method was applied with the use of cadmium. As for the analysis of ammoniacal nitrogen, the modified Berthelot reaction was used.

The organic carbon content was determined using Turin’s method with a heating plate, a Titronic^®^ R 300 digital burette (Xylem Analytrics^®^, Weilheim in Oberbayern, Germany) was used. The soil organic matter (SOM) was evaluated using the weight method with a laboratory balance (Radwag^®^, Radom, Poland), SLW 115 dryer (SciMed^®^, Singapore) and Nabertherm^®^ (Lilienthal, Germany) muffle furnace.

The pH in KCl was determined [94] using a pH-meter CP-505 (Elmetron, Zabrze, Poland), the phosphorus (P_2_O_5_) and potassium content (K_2_O) was evaluated following the Egner–Riehm method [95] using a Sherwood^®^ (Southaven, Mississippi, USA) flame photometer, Thermo Scientific GENESYS^®^ 6 spectrophotometer (Waltham, Massachusetts, USA), and the magnesium content was detected using the Schachtschabel method [96] with a AAS-3 atomic absorption spectrometer.

The biometric parameters of the raspberry roots and plant (stem/leaves) growth were measured. These were the above-ground dry weight (g) of the plant biomass (stems and leaves) and the fresh weight (g) of the plant root biomass. The dry weight was measured after air-drying the plant material at 20 °C for 10 days. Both measurements were made on a laboratory balance (Radwag^®^, Radom, Poland).

### 4.3. Laboratory-Scale Biopreparation Development

#### 4.3.1. *Trichoderma* Sporulation Optimization

The efficiency of spore biomass production under white light was macroscopically assessed on the following commercial ready-to-use agar media: Potato Dextrose Agar (PDA) (A&A Biotechnology^®^, Gdańsk, Poland), Malt Extract Lab Agar (MEA) (Biomaxima^®^, Lublin, Poland), Corn Meal (Biomaxima^®^, Lublin, Poland) with glycerol addition (CM), and laboratory-prepared agar media: modified Mandels and Andreotti with agar (MA), yeast and malt extract minimal agar medium (TR), minimal agar medium with cellulose (MN), soy flour, cellulose, lactose agar medium (MSCL-A) and its liquid version (MSCL-L).

The basic ingredients of apple pomace (Appol^®^, Opole Lubelskie, Poland), wheat bran (Durum, Lubella^®^, Lublin, Poland)) and whey protein concentrate (WPC80, Extensor^®^, Siedlce, Poland) (WBAP) were chosen for the newly developed culture medium for isolates. However, in addition to this basic formula, several other variations were tested in the agar medium version: supplements (WBAP-A), CaCl_2_ and KH_2_PO_4_ (WBAP-B) microcrystalline cellulose and soy flour (WBAP-C), microcrystalline cellulose, soy flour, CaCl_2_ and KH_2_PO_4_ (WBAP-D), for the solid-state medium version these were grounded straw and microcrystalline cellulose (WBAP-F), microcrystalline cellulose (WBAP-G) and sawdust (WBAP-H). The detailed media composition and culture conditions are summarized in Table S2.

To be more specific concerning WBAP-A–WBAP-H, wheat bran had a particle size of 4 × 4 mm, and at least 20% of the particles had a size of 1 × 1 mm and a moisture content in the range of 10–15%. The dried apple pomace had a granulation size of 1–4 mm in agar media and 1–10 mm in solid-state media and a moisture content of 7–10%. The whey protein concentrate (WPC 80, Extensor) had the following amino acid composition: from 1.5% to 3% tyrosine, phenylalanine, histidine, glycine, arginine, from 4% to 5% valine, serine, proline, isoleucine, alanine, and >5% threonine, lysine, leucine, glutamic acid and aspartic acid.

The average spore number per plate was calculated using Thoma’s counting chamber (Blaubrand^®^, Wertheim, Germany) for 90-mm Petri plates with the most excellent sporulation, respectively, for each isolate. The spore biomass from the sporulation experiments was collected, dried at 30 °C, and weighed. Then the number of spores per 1 g of such dry weight was assessed using a Thoma’s cell counting chamber (Blaubrand®, Wertheim, Germany). This allowed for the management of the number of spores added to the construct of the biopreparation formulations.

#### 4.3.2. Solid Formulations—Powder and Pellet

Solid powder formulation (S-PW) and its pelleting modification (S-PE) were proposed. To obtain 1 kg of solid powder formulation (S-PW) of biopreparation for raspberry naturalization, 269 g of wheat bran (Durum, Lubella^®^, Lublin, Poland), 538 g of dried and ground/not ground apple pomace (Appol^®^, Opole Lubelskie, Poland), and 193 g of whey protein concentrate were mixed. Following that, prebiotic supplements such as adenosine, adonitol (Acros Organics^®^, Waltham, Massachusetts, USA), arabitol (Apollo Scientific^®^ Cheshire, United Kingdom), i-erythritol (Vivo^®^, Częstochowa, Poland), mannitol (Merck^®^, Darmstadt, Germany), sorbitol (Merck^®^, Darmstadt, Germany) were added in an amount representing 1% of the rest of the solid ingredients (1.66 g/kg each). Next, the spores were added in an appropriate quantity to obtain a final concentration of 10^8^ spores per 1 g S-PW (equal amount for each isolate of *Trichoderma* sp.) and this was confirmed followed by the total number of fungi test (CFU/g) using a serial dilution of the proposed formulation on a Petri plate (90 mm) containing PDA medium with antibiotics addition (streptomycin and chlortetracycline).

Pellet formulation (S-PE) preparation included two steps. The first one was as described above for solid powder formulation preparation. Subsequently, the second step included the addition of 5% cold-pressed rapeseed oil (Barwy Zdrowia^®^, Tarnogród, Poland) and water addition (10%, 20%, 30%, 40%) was also tested. After that, the material was subjected to pelleting using a hand mincer to select the most promising composition for the subsequent commercial pelleting process. The 3-cm pellets (3 g) were obtained, pellet decompositions in 200 mL of water at 90 rpm were tested to determine the most promising options. This procedure did not include spore addition.

Next, one option of the pellet composition described above (with 30% water addition) with *Trichoderma*-spores addition (10^8^ CFU/g) subjected to one and two stages of pelleting (1st and 2nd variant, respectively). The process was performed in the Skotan^®^ (Chorzów, Poland). It was carried out using a pelleting machine produced by Nawrocki Pelleting Technology^®^ (Żnin, Poland), GRV340 model. The pelleting speed was selected on an ongoing basis so as not to overload the device (to avoid increasing the current intensity above 34 A). The pellet cutting knives were set to the pellet length which was equal to 1 cm. Firstly, the mixture was poured into a mixing hopper equipped with a paddle agitator.

The material inlet temperature was 24.6 °C, material moisture: 4.35–4.52%. Next, oil was added in the amount of 0.5 L to the mixture and short mixing (180 s 3 min^−1^) was performed. Thirdly, the commencement of pelleting was implemented. The 1st variant material inlet temperature was 24.2 °C, the material temperature after the first passage was 58.5 °C, the parameter of the pellet mill was 24 A. For the 2nd variant material the inlet temperature was 25.5 °C, the material temperature after the first passage was 60.3 °C, the electric current of the pellet mill was 26A, the material inlet temperature (second passage) was 58.4 °C, the material temperature after the second passage was 72.7 °C, the electric current of the pellet mill was 25A. The 3 g pellet samples were submitted for decomposition analyses in 200 mL of water at 90 rpm.

#### 4.3.3. Dissolvable Formulations—Powder and Gel

A dissolvable powder formulation (D-PW) and its gel modification (D-GE) were proposed. To obtain 1 kg of dissolvable powder formulation (D-PW), low sugar maltodextrin (Tomex^®^, Biała Podlaska, Poland) was mixed with an M9 medium (Sigma-Aldrich^®^, Saint Louis, Missouri, USA) in a weight relationship of 3:1. Next, a prebiotic supplement mixture (1% in total) was added as described in detail by the solid formulation section.

To screen possible gel formulations (D-GE) the addition of the following gelling agents to the powder formulation were tested: guar gum (AGRO GUMS^®^, Ahmadabad, India), citrus pectin (Cargill^®^, Wayzata, Minnesota, USA)), xanthan gum (Roth^®^, O’Fallon, Illinois, USA)), sodium polyacrylate (hydrogel) (HRT^®^, Kołobrzeg, Poland), carboxymethylcellulose (Sigma-Aldrich^®^ Saint Louis, Missouri, USA), sodium alginate (Biomus^®^, Lublin, Poland)), locust bean gum (Donauchem^®^, Rokietnica, Poland), apple pectin (CPKelco^®^, Großenbrode, Germany). Those plant-derived hydrocolloid bioimmobilization processes were chosen following Waszkiewicz-Robak and Świderski [97].

Guar gum and citrus pectin were tested in the very first step over a wide range of proportions of maltodextrin (0.5%., 1%. 2%, 4%, 6%) and each at different water concentrations (1%, 5%, 10%, 15%, 20%) at room temperature. The second step covered the testing of xanthan gum, carboxymethylcellulose, sodium alginate, locust bean gum (0.5%, 2%, 6%) each at different water concentrations (1%, 10%, 20%). Finally, apple pectin was tested (15% and 20% vs. 15% and 20% of water concentration).

The gel formulation viscosity was measured with a viscometer (VISCOR^®^, North York, Ontario, Canada). The measurement was carried out using an A1S stirrer at 250 rpm. The gel formulation mass that adheres to the raspberry root was tested (root mass 3.93 g).

### 4.4. Statistical Analyses

An analysis of variance (ANOVA) with mean comparisons between treatments was used with Tukey’s post hoc honestly significant differences (HSD) at *p* < 0.05. Statistica 13.1 software (StatSoft^®^, Tulsa, Oklahoma, USA) was used.

## 5. Conclusions

The development of eco-friendly solutions for raspberry production seems to be required since its market is quite significant on a global scale. Bioproducts based on beneficial and antagonistic microorganisms are in line with the long-term policy of the so-called new green order following the global environment directive. It is especially the case with raspberry producers as they struggle with the problem of infection with fungi such as *Botrytis*, *Verticillium*, *Colletotrichum*, and *Phytophthora* fungus-like microorganisms.

As intended, we managed to compose a fungal consortium that was effective against the pathogens *Botrytis cinerea*, *Verticillium* sp. *Colletotrichum acutatum* and *Phytophthora* sp. representatives and efficiently circulated organic matter. The rhizosphere, rhizoplane and roots of wild raspberries were a valuable source of beneficial fungal isolates. Among these, *Trichoderma* isolates G61/18, G63/18, G64/18, G65/18, G67/18, G69/18, G70/18, G78/18, G109/18, G379/18, and G398/18 were finally selected for the prospective consortium. Particular isolates were proven not to interact antagonistically but rather to manifest reasonable antagonistic properties against the aforementioned pathogens and demonstrate a wide range of hydrolytic properties (cellulases, carboxymethylcellulases, *β*-glucosidase, xylanase, protease, amylase). Surely, *Trichoderma* representatives are probably the best-known antagonists, but following the latest trends, we consider representatives of natural counterpart habitats to be suitable. Such an approach, following certain ecological relationships, is intended to contribute to the more effective acclimatization of selected microbes at the targeted location. This so-called naturalization concept which takes into consideration the environmental dependencies of isolates and their resulting properties and adaptability to particular habitats was extended with the testing of a new concept which is the biopreparation of prebiotic supplementation. Prebiotic additive supplementation with a mixture of adonitol, arabitol, erythritol, mannitol, sorbitol, and adenosine were proven in a laboratory experiment to be efficient in stimulating the growth of selected *Trichoderma* isolates, and further pot and field experiments are planned to meet the expectation that antagonistic isolates develop better than pathogens, in the presence of prebiotics also in field conditions.

Secondly, we sought the positive influence of the methodically chosen *Trichoderma*-based consortium on raspberry plants and soil-associated properties. Thus, a raspberry pot experiment was set up to take advantage of *Botrytis*, *Verticillium*, *Colletotrichum*, *Phytophthora* pathosystems and different *Trichoderma*-protection strategies, such as root inoculations and watering. The certain correspondence of the effectiveness of the naturalization strategy at least in terms of the aboveground biomass increase as an initial effect, with the type of pathogen being noted, especially for *Botrytis* sp., *Colletotrichum* sp. and *Verticillium* sp. Nevertheless, we are aware that plant biomass production changes do not reflect the entire and even possible *Trichoderma* sp.–pathogen–raspberry relationship, especially since various factors influence this bond. Thus, future, long-term experiments are planned, including the evaluation, for example, fruit yield, soil enzymatic activity, a summary of the presence of pathogens, the plant growth-promoting microbiome network, or simply the symptoms of diseases caused by those pathogens. The obtained results corresponded to a positive *Trichoderma*-based consortium early response and may encourage further activities in this area.

Thirdly, the development of biopreparations targeted at raspberry naturalization was shown. This proposal included an assortment of the *Trichoderma* spp. culture medium compositions and carriers. The medium was established on easily accessible waste, namely apple pomace and wheat bran, with protein concentrate addition as a source of amino acids. It was proven that *Trichoderma* isolates grow and sporulate successfully on media that includes those ingredients, however, they require a suitable formulation matched to particular isolates. Thus, isolates G64/18 and G70/18 only cope well with apple pomace, wheat bran and whey protein, and therefore they do not require additional ingredients in their media to sporulate well. Isolates G63/18 and G65/18 require microcrystalline cellulose addition. In turn, G69/18, G78/18, and G109/18 need a straw and microcrystalline cellulose addition. 

Finally, G61/18 needs some easily digestible forms of carbon (adonitol, arabitol, erythritol, glycerol, mannitol, and sorbitol). Isolates G379/18 and G398/18 require further optimization. What is more, a particular motivation factor in this study was the formulation of concepts leading to the development of tolerably different practical application methods of the *Trichoderma*-based consortium. Therefore, apple pomace and wheat bran with rapeseed oil and 30% water content were denoted as comprising suitable properties for the formation of pellets. It has also been proven in this research that maltodextrin may serve as an insoluble carrier. Xanthan gum, carboxymethylcellulose, guar gum, or apple pectin with adequate water addition is also efficient in *Trichoderma* spp. gel-entrapment, and unequivocally demonstrates viscosity at a level that is sufficient to stick to the raspberry root in such a gel form. 

The undertaken commitment comprises screening criteria efficacy, fungal and targeted disease ecology issues, waste utilization and may lead to a prospective production scale.

## Figures and Tables

**Table 1:** The factsheet concerning the wild raspberry fungal isolate and pathogen collections used, including the data of their collection, identification, and GenBank submission sequences. Abbreviations: Isolate code according to the Institute of Agrophysics, Polish Academy of Sciences—IA PAS, Laboratory of Environmental and Molecular Microbiology; Sabouraud—Sabouraud Dextrose Agar (Biocorp^®^, Warszawa, Poland); Rose Bengal—Rose Bengal Agar with Chloramphenicol (Biocorp^®^, Warszawa, Poland); Pikovskaya—Pikovskaya Agar Medium; Potato Dextrose—Potato Dextrose Lab Agar (A&A Biotechnology^®^, Gdańsk, Poland).

Isolate Code	GenBank Sequences Accession Number	Identification	Isolation Compartment and Institution	GPS Coordinates	Forest District	Agar Medium
G25/18	MW151041	*Apiotrichum porosum*	Wild raspberry rhizosphere, IA PAS, Poland	51°54’46.6” N 22°28’29.9” E	Świdnik	Sabouraud
G26/18	MW151042	*Apiotrichum porosum*	Wild raspberry rhizosphere, IA PAS, Poland	-	Łuków	Sabouraud
G27/18	MW151043	*Cryptococcus* sp.	Wild raspberry rhizosphere, IA PAS, Poland	51°01’08.3” N 22°01’57.8” E	Kraśnik	Rose Bengal
G28/18	MW151044	*Aureobasidium* sp.	Wild raspberry rhizosphere, IA PAS, Poland	51°17’13.7” N 21°95’99.3” E	Kraśnik	Rose Bengal
G29/18	-	-	Wild raspberry roots, IA PAS, Poland	51°01’08.3” N 22°01’57.8” E	Kraśnik	Sabouraud
G30/18	MW150994.1	*Trichoderma* sp.	Wild raspberry roots, IA PAS, Poland	51°00’90.3” N 22°01’13.9” E	Kraśnik	Rose Bengal
G31/18	MW150994	*Trichoderma* sp.	Wild raspberry roots, IA PAS, Poland	51°41’39.5” N 22°24’40.2” E	Łuków	Rose Bengal
G32/18	MW150995	*Apiotrichum porosum*	Wild raspberry roots, IA PAS, Poland	51°00’93.6” N 22°01’22.0” E	Kraśnik	Rose Bengal
G33/18	MW151045	*Solicoccozyma* sp.	Wild raspberry rhizosphere, IA PAS, Poland	-	Łuków	Sabouraud
G34/18	MW151046	*Apiotrichum porosum*	Wild raspberry rhizosphere, IA PAS, Poland	51°54’46.6” N 22°28’29.9” E	Świdnik	Sabouraud
G35/18	MW151047	*Cryptococcus* sp.	Wild raspberry rhizosphere, IA PAS, Poland	51°17’13.0” N 21°95’99.7” E	Kraśnik	Sabouraud
G36/18	MW151048	*Solicoccozyma* sp.	Wild raspberry rhizosphere, IA PAS, Poland	51°54’47.9” N 22°28’44.8” E	Świdnik	Sabouraud
G37/18	MW151049	*Solicoccozyma* sp.	Wild raspberry rhizosphere, IA PAS, Poland	51°17’13.7” N 21°95’99.3” E	Kraśnik	Sabouraud
G38/18	MW151050	*Solicoccozyma* sp.	Wild raspberry rhizosphere, IA PAS, Poland	51°17’13.7” N 21°95’99.3” E	Kraśnik	Sabouraud
G39/18	MW151051	*Umbelopsis vinacea*	Wild raspberry rhizosphere, IA PAS, Poland	51°01’08.3” N 22°01’77.8” E	Kraśnik	Sabouraud
G40/18	-	-	Wild raspberry rhizosphere, IA PAS, Poland	51°54’46.6” N 22°28’29.9” E	Świdnik	Sabouraud
G41/18	MW150996	*Umbelopsis* sp.	Wild raspberry roots, IA PAS, Poland	51°79’04.0” N 22°20’68.1” E	Łuków	Sabouraud
G42/18	MW151052	*Umbelopsis isabelina*	Wild raspberry rhizosphere, IA PAS, Poland	-	Siedlce	Sabouraud
G43/18	MW151053	*Apiotrichum porosum*	Wild raspberry rhizosphere, IA PAS, Poland	51°17’13.0” N 21°95’99.7” E	Kraśnik	Rose Bengal
G44/18	MW151054	*Apiotrichum porosum*	Wild raspberry rhizosphere, IA PAS, Poland	51°54’46.6” N 22°28’29.9” E	Świdnik	Rose Bengal
G45/18	MW151055	*Mortierella* sp.	Wild raspberry rhizosphere, IA PAS, Poland	51°00’90.3” N 22°01’13.9” E	Kraśnik	Sabouraud
G46/18	MW151056	*Umbelopsis* sp.	Wild raspberry rhizosphere, IA PAS, Poland	-	Siedlce	Sabouraud
G47/18	MW151057	*Umbelopsis* sp.	Wild raspberry rhizosphere, IA PAS, Poland	-	Łuków	Sabouraud
G48/18	-	-	Wild raspberry rhizosphere, IA PAS, Poland	51°01’08.3” N 22°01’77.8” E	Kraśnik	Sabouraud
G49/18	MW151058	*Apiotrichum porosum*	Wild raspberry rhizosphere, IA PAS, Poland	-	Łuków	Sabouraud
G50/18	MW151059	*Apiotrichum porosum*	Wild raspberry rhizosphere, IA PAS, Poland	51°54’47.9” N 22°28’44.8” E	Świdnik	Sabouraud
G51/18	MW151060	*Geotrichum europaeum*	Wild raspberry rhizosphere, IA PAS, Poland	50°39’27.5” N 22°24’36.6” E	Janów Lubelski	Sabouraud
G52/18	MW151061	*Geotrichum europaeum*	Wild raspberry rhizosphere, IA PAS, Poland	50°39’42.1” N 22°24’21.0” E	Janów Lubelski	Rose Bengal
G53/18	MW151062	*Apiotrichum porosum*	Wild raspberry rhizosphere, IA PAS, Poland	51°54’46.6” N 22°28’29.9” E	Świdnik	Rose Bengal
G54/18	MW151063	*Plectospahrella cucumerina*	Wild raspberry rhizosphere, IA PAS, Poland	-	Janów Lubelski	Sabouraud
G55/18	MW257185	*Plectospahrella* sp.	Wild raspberry rhizosphere, IA PAS, Poland	-	Janów Lubelski	Sabouraud
G56/18	MW257186	*Trichoderma* sp.	Wild raspberry rhizosphere, IA PAS, Poland	51°54’47.9” N 22°28’44.8” E	Świdnik	Sabouraud
G57/18	-	-	Wild raspberry rhizosphere, IA PAS, Poland	-	Siedlce	Sabouraud
G58/18	-	-	Wild raspberry rhizosphere, IA PAS, Poland	51°17’10.0” N 21°95’99.8” E	Kraśnik	Sabouraud
G59/18	-	-	Wild raspberry roots, IA PAS, Poland	51°00’90.3” N 22°01’13.9” E	Kraśnik	Rose Bengal
G60/18	-	-	Wild raspberry rhizosphere, IA PAS, Poland	50°39’27.5” N 22°24’36.6” E	Janów Lubelski	Rose Bengal
G61/18	MW233576	*Trichoderma* sp.	Wild raspberry rhizosphere, IA PAS, Poland		Siedlce	Rose Bengal
G62/18	-	-	Wild raspberry roots, IA PAS, Poland	51°01’05.1” N 22°01’67.2” E	Kraśnik	Rose Bengal
G63/18	MT558561	*Trichoderma* sp.	Wild raspberry roots, IA PAS, Poland	51°54’46.2” N 22°28’30.6” E	Świdnik	Rose Bengal
G64/18	MT558562	*Trichoderma* sp.	Wild raspberry roots, IA PAS, Poland	51°54’46.6” N 22°28’29.9” E	Świdnik	Sabouraud
G65/18	MW233577	*Trichoderma* sp.	Wild raspberry rhizosphere, IA PAS, Poland	51°54’46.2” N 22°28’30.6” E	Świdnik	Rose Bengal
G66/18	-	-	Wild raspberry rhizosphere, IA PAS, Poland	-	Janów Lubelski	Rose Bengal
G67/18	MW205828	*Trichoderma* sp.	Wild raspberry roots, IA PAS, Poland	51°54’47.9” N 22°28’44.8” E	Świdnik	Rose Bengal
G68/18	-	-	Wild raspberry rhizosphere, IA PAS, Poland	-	Siedlce	Sabouraud
G69/18	MT559294	*Trichoderma* sp.	Wild raspberry rhizosphere, IA PAS, Poland	51°54’47.9” N 22°28’44.8” E	Świdnik	Rose Bengal
G70/18	MW233578	*Trichoderma* sp.	Wild raspberry rhizosphere, IA PAS, Poland	51°54’47.9” N 22°28’44.8” E	Świdnik	Rose Bengal
G71/18	-	-	Wild raspberry rhizosphere, IA PAS, Poland		Janów Lubelski	Rose Bengal
G72/18	-	-	Wild raspberry rhizosphere, IA PAS, Poland	-	Siedlce	Rose Bengal
G73/18	-	-	Wild raspberry rhizosphere, IA PAS, Poland	51°17’13.0” N 21°95’99.7” E	Kraśnik	Sabouraud
G74/18	-	-	Wild raspberry rhizosphere, IA PAS, Poland	51°17’13.0” N 21°95’99.7” E	Kraśnik	Rose Bengal
G75/18	MT558563.1	*Trichoderma* sp.	Wild raspberry rhizosphere, IA PAS, Poland	51°54’47.9” N 22°28’44.8” E	Świdnik	Rose Bengal
G76/18	MW257187	*Trichoderma* sp.	Wild raspberry rhizosphere, IA PAS, Poland	51°17’13.7” N 21°95’99.3” E	Kraśnik	Rose Bengal
G77/18	MW257194	*Trichoderma* sp.	Wild raspberry roots, IA PAS, Poland	50°39’27.3” N 22°24’10.7” E	Janów Lubelski	Sabouraud
G78/18	MW205829	*Trichoderma* sp.	Wild raspberry roots, IA PAS, Poland	50°39’27.3” N 22°24’10.7” E	Janów Lubelski	Rose Bengal
G79/18	-	-	Wild raspberry roots, IA PAS, Poland	51°17’13.0” N 21°95’99.7” E	Kraśnik	Rose Bengal
G80/18	-	-	Wild raspberry rhizosphere, IA PAS, Poland	51°17’13.0” N 21°95’99.7” E	Kraśnik	Rose Bengal
G81/18	MW257188	*Mortierella* sp.	Wild raspberry rhizosphere, IA PAS, Poland	-	Kraśnik	Rose Bengal
G83/18	MW257189	*Mortierella* sp.	Wild raspberry rhizosphere, IA PAS, Poland	-	Janów Lubelski	Rose Bengal
G84/18	-	-	Wild raspberry roots, IA PAS, Poland	51°17’10.0” N 21°95’99.8” E	Kraśnik	Sabouraud
G85/18	-	-	Wild raspberry rhizosphere, IA PAS, Poland	-	Janów Lubelski	Sabouraud
G86/18	-	-	Wild raspberry rhizosphere, IA PAS, Poland	-	Janów Lubelski	Rose Bengal
G87/18	-	-	Wild raspberry rhizosphere, IA PAS, Poland	51°00’93.6” N 22°01’22.0” E	Kraśnik	Rose Bengal
G88/18	-	-	Wild raspberry roots, IA PAS, Poland	51°00’93.6” N 22°01’22.0” E	Kraśnik	Rose Bengal
G89/18	MW257190	*Mucor* sp.	Wild raspberry rhizosphere, IA PAS, Poland	-	Janów Lubelski	Rose Bengal
G90/18	-	-	Wild raspberry rhizosphere, IA PAS, Poland	51°17’10.0” N 21°95’99.8” E	Kraśnik	Sabouraud
G91/18	MW257191	*Mucor* sp.	Wild raspberry rhizosphere, IA PAS, Poland	51°00’92.6” N 22°01’12.0” E	Kraśnik	Sabouraud
G92/18	MW257192	*Absidia* sp.	Wild raspberry rhizosphere, IA PAS, Poland	51°00’90.3” N 22°01’13.9” E	Kraśnik	Sabouraud
G93/18	MW257193	Mucor sp.	Wild raspberry rhizosphere, IA PAS, Poland	51°00’90.3” N 22°01’13.9” E	Kraśnik	Rose Bengal
G94/18	MW257196	*Fusarium* sp.	Wild raspberry roots, IA PAS, Poland	50°39’27.5” N 22°24’36.6” E	Janów Lubelski	Rose Bengal
G95/18	MW257197	*Fusarium* sp.	Wild raspberry roots, IA PAS, Poland	-	Janów Lubelski	Rose Bengal
G96/18	MW257195	*Umbelopsis* sp.	Wild raspberry roots, IA PAS, Poland	51°41’39.5” N 22°24’40.2” E	Łuków	Sabouraud
G97/18	-	-	Wild raspberry rhizosphere, IA PAS, Poland	50°39’27.3” N 22°24’10.7” E	Janów Lubelski	Rose Bengal
G98/18	-	-	Wild raspberry roots, IA PAS, Poland	51°54’46.6” N 22°28’29.9” E	Świdnik	Sabouraud
G99/18	-	-	Wild raspberry roots, IA PAS, Poland	51°54’46.6” N 22°28’29.9” E	Świdnik	Sabouraud
G100/18	-	-	Wild raspberry rhizosphere, IA PAS, Poland	51°54’46.6” N 22°28’29.9” E	Świdnik	Sabouraud
G101/18	-	-	Wild raspberry rhizosphere, IA PAS, Poland	51°00’90.3” N 22°01’13.9” E	Kraśnik	Sabouraud
G102/18	-	-	Wild raspberry rhizosphere, IA PAS, Poland	51°17’13.0” N 21°95’99.7” E	Kraśnik	Sabouraud
G103/18	-	-	Wild raspberry rhizosphere, IA PAS, Poland	51°54’47.9” N 22°28’44.8” E	Świdnik	Sabouraud
G104/18	-	-	Wild raspberry rhizosphere, IA PAS, Poland	51°00’90.3” N 22°01’13.9” E	Kraśnik	Sabouraud
G105/18	-	-	Wild raspberry roots, IA PAS, Poland		Siedlce	Sabouraud
G106/18	MW150997	*Fusarium venenatum*	Wild raspberry roots, IA PAS, Poland	51°17’13.7” N 21°95’99.3” E	Kraśnik	Sabouraud
G107/18	MW150998	*Fusarium* sp.	Wild raspberry roots, IA PAS, Poland	51°54’46.2” N 22°28’30.6” E	Świdnik	Sabouraud
G108/18	MW150999	*Fusarium roseum*	Wild raspberry roots, IA PAS, Poland	51°17’13.0” N 21°95’99.7” E	Kraśnik	Sabouraud
G109/18	MW233579	*Trichoderma* sp.	Wild raspberry rhizosphere, IA PAS, Poland	51°17’13.7” N 21°95’99.3” E	Kraśnik	Sabouraud
G110/18	MW151000	*Fusarium* sp.	Wild raspberry roots, IA PAS, Poland	50°39’27.3” N 22°24’10.7” E	Janów Lubelski	Sabouraud
G111/18	-	-	Wild raspberry roots, IA PAS, Poland	50°39’42.1” N 22°24’21.0” E	Janów Lubelski	Sabouraud
G112/18	-	-	Wild raspberry roots, IA PAS, Poland	-	Siedlce	Rose Bengal
G113/18	MW151064	*Talaromyces* sp.	Wild raspberry rhizosphere, IA PAS, Poland	-	Siedlce	Sabouraud
G114/18	-	-	Wild raspberry rhizosphere, IA PAS, Poland	-	Janów Lubelski	Rose Bengal
G115/18	MW151001	*Penicillium coprobium*	Wild raspberry roots, IA PAS, Poland	51°17’10.0” N 21°95’99.8” E	Kraśnik	Rose Bengal
G116/18	MW151065	*Penicillium coprobium*	Wild raspberry rhizosphere, IA PAS, Poland	50°39’27.3” N 22°24’10.7” E	Janów Lubelski	Rose Bengal
G117/18	MW151066	*Penicillium* sp.	Wild raspberry rhizosphere, IA PAS, Poland	51°41’39.5” N 22°24’40.2” E	Łuków	Rose Bengal
G118/18	MW151067	*Penicillium* sp.	Wild raspberry rhizosphere, IA PAS, Poland	50°39’27.5” N 22°24’36.6” E	Janów Lubelski	Rose Bengal
G119/18	-	-	Wild raspberry rhizosphere, IA PAS, Poland	51°01’05.1” N 22°01’67.2” E	Kraśnik	Rose Bengal
G120/18	MW151068	*Penicillium* sp.	Wild raspberry rhizosphere, IA PAS, Poland	-	Janów Lubelski	Sabouraud
G121/18	MW151069	*Penicillium* sp.	Wild raspberry rhizosphere, IA PAS, Poland	51°54’46.2” N 22°28’30.6” E	Świdnik	Rose Bengal
G122/18	MW151070	*Penicillium* sp.	Wild raspberry rhizosphere, IA PAS, Poland	51°01’05.1” N 22°01’67.2” E	Kraśnik	Rose Bengal
G123/18	MW151071	*Penicillium* sp.	Wild raspberry rhizosphere, IA PAS, Poland	-	Siedlce	Rose Bengal
G124/18	MW151072	*Penicillium* sp.	Wild raspberry rhizosphere, IA PAS, Poland	51°17’13.0” N 21°95’99.7” E	Kraśnik	Rose Bengal
G125/18	MW151002	*Penicillium* sp.	Wild raspberry roots, IA PAS, Poland	-	Janów Lubelski	Rose Bengal
G126/18	-	-	Wild raspberry rhizosphere, IA PAS, Poland	-	Janów Lubelski	Rose Bengal
G127/18	-	-	Wild raspberry rhizosphere, IA PAS, Poland	51°54’46.6” N 22°28’29.9” E	Świdnik	Rose Bengal
G128/18	MW151073	*Penicillium* sp.	Wild raspberry rhizosphere, IA PAS, Poland	51°54’47.9” N 22°28’44.8” E	Świdnik	Sabouraud
G129/18	MW151074	*Penicillium* sp.	Wild raspberry rhizosphere, IA PAS, Poland		Łuków	Sabouraud
G130/18	-	-	Wild raspberry rhizosphere, IA PAS, Poland	51°54’46.6” N 22°28’29.9” E	Świdnik	Sabouraud
G131/18	MW151075	*Cladosporium* sp.	Wild raspberry rhizosphere, IA PAS, Poland		Łuków	Sabouraud
G132/18	MW151076	*Cladosporium* sp.	Wild raspberry rhizosphere, IA PAS, Poland	50°39’27.5” N 22°24’36.6” E	Janów Lubelski	Rose Bengal
G133/18	MW151077	*Cladosporium* sp.	Wild raspberry rhizosphere, IA PAS, Poland	51°17’10.0” N 21°95’99.8” E	Kraśnik	Sabouraud
G134/18	MW151078	*Humicola* sp.	Wild raspberry rhizosphere, IA PAS, Poland	51°17’13.0” N 21°95’99.7” E	Kraśnik	Rose Bengal
G135/18	MW151079	*Penicillium* sp.	Wild raspberry rhizosphere, IA PAS, Poland	-	Janów Lubelski	Sabouraud
G136/18	MW151080	*Massarina* sp.	Wild raspberry rhizosphere, IA PAS, Poland	-	Janów Lubelski	Sabouraud
G137/18	MW151081	*Cladosporium* sp.	Wild raspberry rhizosphere, IA PAS, Poland	-	Janów Lubelski	Sabouraud
G138/18	MW151082	*Cladosporium* sp.	Wild raspberry rhizosphere, IA PAS, Poland	51°17’13.0” N 21°95’99.7” E	Kraśnik	Sabouraud
G139/18	MW151083	*Penicillium* sp.	Wild raspberry rhizosphere, IA PAS, Poland	-	Siedlce	Sabouraud
G140/18	MW250233	*Penicillium* sp.	Wild raspberry rhizosphere, IA PAS, Poland	50°39’27.3” N 22°24’10.7” E	Janów Lubelski	Rose Bengal
G141/18	-	-	Wild raspberry roots, IA PAS, Poland	50°39’27.3” N 22°24’10.7” E	Janów Lubelski	Rose Bengal
G142/18	MW151003	*Alternaria* sp.	Wild raspberry roots, IA PAS, Poland	51°17’10.0” N 21°95’99.8” E	Kraśnik	Sabouraud
G143/18	-	-	Wild raspberry roots, IA PAS, Poland	51°17’13.0” N 21°95’99.7” E	Kraśnik	Sabouraud
G144/18	MW151004	*Mucor moelleri*	Wild raspberry roots, IA PAS, Poland	51°01’05.1” N 22°01’67.2” E	Kraśnik	Sabouraud
G145/18	MW151005	*Podospora* sp.	Wild raspberry roots, IA PAS, Poland	50°39’42.1” N 22°24’21.0” E	Janów Lubelski	Sabouraud
G146/18	MW151006	*Apiosporaceae*	Wild raspberry roots, IA PAS, Poland	50°39’42.1” N 22°24’21.0” E	Janów Lubelski	Sabouraud
G147/18	MW151084	*Coniothyrium* sp.	Wild raspberry rhizosphere, IA PAS, Poland	50°39’42.1” N 22°24’21.0” E	Janów Lubelski	Rose Bengal
G148/18	MW151085	*Coniothyrium* sp.	Wild raspberry rhizosphere, IA PAS, Poland	-	Janów Lubelski	Rose Bengal
G149/18	MW151086	*Penicillium* sp.	Wild raspberry rhizosphere, IA PAS, Poland	51°17’13.0” N 21°95’99.7” E	Kraśnik	Sabouraud
G150/18	MW151087	*Penicillium* sp.	Wild raspberry rhizosphere, IA PAS, Poland	-	Janów Lubelski	Sabouraud
G151/18	MW151088	*Pyrenochaeta* sp.	Wild raspberry rhizosphere, IA PAS, Poland	-	Janów Lubelski	Rose Bengal
G152/18	MW151089	*Penicillium* sp.	Wild raspberry rhizosphere, IA PAS, Poland	-	Siedlce	Rose Bengal
G153/18	MW151090	*Penicillium* sp.	Wild raspberry rhizosphere, IA PAS, Poland	-	Siedlce	Sabouraud
G154/18	MW151091	*Fusarium* sp.	Wild raspberry rhizosphere, IA PAS, Poland	-	Janów Lubelski	Sabouraud
G155/18	-	-	Wild raspberry rhizosphere, IA PAS, Poland	-	Siedlce	Rose Bengal
G157/18	MW151007	Mucor sp.	Wild raspberry roots, IA PAS, Poland	50°39’42.1” N 22°24’21.0” E	Janów Lubelski	Sabouraud
G158/18	-	-	Wild raspberry rhizosphere, IA PAS, Poland	-	Siedlce	Rose Bengal
G159/18	-	-	Wild raspberry rhizosphere, IA PAS, Poland	51°54’46.6” N 22°28’29.9” E	Świdnik	Sabouraud
G375/18	-	-	Wild raspberry rhizosphere, IA PAS, Poland	-	Janów Lubelski	Pikovskaya
G376/18	-	-	Wild raspberry rhizosphere, IA PAS, Poland	50°39’42.1” N 22°24’21.0” E	Janów Lubelski	Pikovskaya
G377/18	-	-	Wild raspberry rhizosphere, IA PAS, Poland	-	Janów Lubelski	Pikovskaya
G378/18	-	-	Wild raspberry rhizosphere, IA PAS, Poland	-	Janów Lubelski	Pikovskaya
G379/18	MT559285	*Trichoderma* sp.	Wild raspberry rhizosphere, IA PAS, Poland	50°39’42.1” N 22°24’21.0” E	Janów Lubelski	Pikovskaya
G380/18	-	-	Wild raspberry rhizosphere, IA PAS, Poland	50°39’42.1” N 22°24’21.0” E	Janów Lubelski	Pikovskaya
G381/18	-	-	Wild raspberry rhizosphere, IA PAS, Poland	50°39’42.1” N 22°24’21.0” E	Janów Lubelski	Pikovskaya
G382/18	-	-	Wild raspberry rhizosphere, IA PAS, Poland	-	Janów Lubelski	Pikovskaya
G383/18	-	-	Wild raspberry rhizosphere, IA PAS, Poland	-	Janów Lubelski	Pikovskaya
G384/18	-	-	Wild raspberry rhizosphere, IA PAS, Poland	-	Kraśnik	Pikovskaya
G385/18	-	-	Wild raspberry rhizosphere, IA PAS, Poland	-	Kraśnik	Pikovskaya
G386/18	-	-	Wild raspberry rhizosphere, IA PAS, Poland	-	Kraśnik	Pikovskaya
G387/18	-	-	Wild raspberry rhizosphere, IA PAS, Poland	-	Kraśnik	Pikovskaya
G388/18	-	-	Wild raspberry rhizosphere, IA PAS, Poland	-	Kraśnik	Pikovskaya
G389/18	-	-	Wild raspberry rhizosphere, IA PAS, Poland	-	Janów Lubelski	Pikovskaya
G390/18	-	-	Wild raspberry rhizosphere, IA PAS, Poland	-	Janów Lubelski	Pikovskaya
G391/18	-	-	Wild raspberry rhizosphere, IA PAS, Poland	-	Janów Lubelski	Pikovskaya
G392/18	-	-	Wild raspberry rhizosphere, IA PAS, Poland	-	Janów Lubelski	Pikovskaya
G393/18	-	-	Wild raspberry rhizosphere, IA PAS, Poland	-	Janów Lubelski	Pikovskaya
G394/18	-	-	Wild raspberry rhizosphere, IA PAS, Poland	-	Janów Lubelski	Pikovskaya
G395/18	-	-	Wild raspberry rhizosphere, IA PAS, Poland	-	Kraśnik	Pikovskaya
G396/18	-	-	Wild raspberry rhizosphere, IA PAS, Poland	-	Kraśnik	Pikovskaya
G397/18	-	-	Wild raspberry rhizosphere, IA PAS, Poland	-	Kraśnik	Pikovskaya
G398/18	MT559286	*Trichoderma* sp.	Wild raspberry rhizosphere, IA PAS, Poland	50°39’42.1” N 22°24’21.0” E	Janów Lubelski	Pikovskaya
G399/18	-	-	Wild raspberry rhizosphere, IA PAS, Poland	-	Kraśnik	Pikovskaya
G6/19	-	-	Wild raspberry rhizosphere, IA PAS, Poland	50°39’42.1” N 22°24’21.0” E	Janów Lubelski	Potato Dextrose
G172/18	MT126803	*Colletotrichum* sp.	Strawberry fruit, IA PAS, Poland	-	-	Potato Dextrose
G371/18	MT558572.1	*Colletotrichum* sp.	Research Institute of Horticulture	-	-	Potato Dextrose
G166/18	MT126798	*Colletotrichum* sp.	Strawberry roots, IA PAS, Poland	-	-	Potato Dextrose
G293/18	MT133324	*Verticillium* sp.	Strawberry roots, IA PAS, Poland	-	-	Potato Dextrose
G296/18	MT133320	*Verticillium* sp.	Strawberry roots, IA PAS, Poland	-	-	Potato Dextrose
G297/18	MT133316	*Verticillium* sp.	Strawberry roots, IA PAS, Poland	-	-	Potato Dextrose
G368/18	MT558571	*Phytophthora* sp.	Research Institute of Horticulture	-	-	-
G408/18	MT126670	*Phytophthora* sp.	Strawberry roots, IA PAS, Poland	-	-	Potato Dextrose
G373/18	-	*Phytophthora* sp.	Research Institute of Horticulture	-	-	-
G369/18	MT558729	*Phytophthora cactorum*	Research Institute of Horticulture	-	-	-
G275/16	MT154302	*Botrytis* sp.	Strawberry roots, IA PAS, Poland	-	-	Potato Dextrose
G277/18	MT154304	*Botrytis* sp.	Strawberry roots, IA PAS, Poland	-	-	Potato Dextrose
G276/18	MT154303	*Botrytis* sp.	Strawberry roots, IA PAS, Poland	-	-	Potato Dextrose

**Table 2:** The antagonistic properties of *Trichoderma* isolates against phytopathogens resulting from *Botrytis* spp., *Colletotrichum* spp., *Verticillium* spp., *Phytophthora* spp. implied as diameters of growth and/or sporulation inhibition in an experiment on Petri dishes. Standard errors are provided, *n* = 3. Abbreviations: “-” indicate no growth or sporulation inhibition noted.

*Trichoderma* spp.	*Botrytis* spp.			*Colletotrichum* spp.			*Verticillium* spp.			*Phytophthora* spp.		
		Growth Inhibition (mm)	Sporulation Inhibition (mm)		Growth Inhibition (mm)	Sporulation Inhibition (mm)		Growth Inhibition (mm)	Sporulation Inhibition (mm)		Growth Inhibition (mm)	Sporulation Inhibition (mm)
G61/18	G275/18	46.5 ± 3.5	-	G172/18	46.7 ± 18.1	-	G293/18	22.5 ± 1.6	70.6 ± 3.8	G368/18	-	49.3 ± 9.4
G63/18		63.9 ± 5.5	-		45.5 ± 8.0	-		44.2 ± 13.0	67.5 ± 1.2		33.5 ± 0.0	-
G64/18		69.9 ± 4.9	-		45.9 ± 2.6	-		42.3 ± 5.1	72.9 ± 3.8		21.9 ± 1.8	55.7 ± 2.4
G65/18		39.0 ± 8.5	-		32.1 ± 1.8	-		24.2 ± 1.2	55.8 ± 1.8		12.7 ± 0.6	-
G67/18		60.4 ± 13.5	-		22.3 ± 2.0	-		47.6 ± 10.3	-		24.7 ± 3.1	-
G69/18		51.0 ± 15.6	-		38.3 ± 7.0	-		34.7 ± 1.8	63.4 ± 7.3		29.7 ± 1.4	58.8 ± 10.8
G70/18		32.5 ± 7.8	-		23.9 ± 4.7	-		40.0 ± 0.0	-		90.0 ± 0.0	-
G78/18		45.6 ± 15.1	-		31.3 ± 1.8	-		20.7 ± 0.6	62.4 ± 1.1		37.6 ± 9.3	-
G109/18		38.1 ± 4.5	-		17.1 ± 1.2	-		53.0 ± 13.2	-		67.9 ± 14.7	-
G379/18		40.1 ± 1.4	-		-	18.0 ± 0.0		40.5 ± 3.5	-		-	34.4 ± 2.3
G398/18		38.0 ± 1.9	-		-	24.0 ± 2.8		30.0 ± 1.4	55.6 ± 3.4		-	-
G61/18	G277/18	66.8 ± 16.4	-	G371/18	58.9 ± 6.7	-	G296/18	23.4 ± 4.4	66.6 ± 8.1	G373/18	48.3 ± 1.3	-
G63/18		52.8 ± 15.6	-		38.7 ± 5.9	-		33.8 ± 8.6	72.3 ± 11.6		48.3 ± 9.4	-
G64/18		60.4 ± 15.9	-		50.4 ± 11.5	-		36.9 ± 9.7	63.4 ± 6.1		54.2 ± 16.4	-
G65/18		55.2 ± 11.2	-		36.8 ± 12.7	-		25.3 ± 0.6	59.7 ± 5.2		39.4 ± 8.0	-
G67/18		22.5 ± 0.7	54.0 ± 9.9		29.2 ± 2.2	-		55.1 ± 8.9	-		25.7 ± 3.9	66.6 ± 14.9
G69/18		39.6 ± 5.5	-		27.8 ± 6.8	-		37.4 ± 12.1	68.0 ± 10.8		49.0 ± 21.2	-
G70/18		38.7 ± 5.5	-		20.1 ± 3.0	-		37.6 ± 0.6	-		37.7 ± 10.9	-
G78/18		37.0 ± 1.4	-		36.2 ± 5.9	-		21.4 ± 0.6	56.8 ± 10.1		39.7 ± 1.4	-
G109/18		29.1 ± 8.0	-		15.3 ± 7.2	-		27.8 ± 9.3	-		50.2 ± 4.0	-
G379/18		36.1 ± 2.2	-		-	23.0 ± 1.4		-	55.0 ± 15.6		-	49.6 ± 1.3
G398/18		30.0 ± 2.8	-		-	16.0 ± 4.8		31.2 ± 0.7	49.6 ± 3.6		-	56.7 ± 12.7
G61/18	G276/18	40.2 ± 9.0	-	G166/18	90.0 ±0.0		G297/18	29.0 ± 2.2	73.2 ± 12.7	G369/18	54.9 ± 12.1	-
G63/18		31.2 ± 2.5	70.8 ± 6.9		45.8 ± 20.8	-		47.1 ± 6.1	79.3 ± 15.2		35.2 ± 0.0	-
G64/18		71.5 ± 17.0	-		64.0 ± 13.1	-		55.6 ± 12.3	83.6 ± 9.1		20.5 ± 2.9	62.4 ± 0.0
G65/18		35.5 ± 2.0	55.9 ± 4.0		30.1 ± 1.8	-		-	65.8 ± 14.2		14.2 ± 0.5	-
G67/18		14.5 ± 3.3	38.4 ± 27.8		33.0 ± 5.5	-		61.8 ± 4.4	-		25.9 ± 3.1	-
G69/18		33.5 ± 3.4	61.3 ± 4.5		27.0 ±0.0	-		37.1 ± 2.7	60.0 ± 8.5		30.3 ± 1.5	76.4 ± 1.5
G70/18		31.3 ± 4.0	-		46.7 ± 30.6	-		40.7 ± 0.6	-		90.0 ± 0.0	
G78/18		34.4 ± 14.9	-		25.8 ± 3.5	-		24.7 ± 0.0	56.2 ± 1.7		61.0 ± 13.3	-
G109/18		34.7 ± 13.1	-		19.4 ± 3.3	-		25.8 ± 5.9	-		83.5 ± 9.2	-
G379/18		-	44.5 ± 0.0		-	23.04 ± 5.3		63.1 ± 11.7	-		-	48.2 ± 0.0
G398/18		-	47.4 ± 6.7		-	28.08 ± 0.0		-	69.3 ± 8.9		-	14.02 ± 1.3

**Table 3:** The enzymatic activity of selected *Trichoderma* isolates. Abbreviations: cellulases (FPU), carboxymethylcellulases (CMC), β-glucosidase (BGL), xylanase (XYL), protease (PRO), amylase (AMY). Standard errors are provided, *n* = 3.

Isolate	FPU				CMC				BGL	XYL	PRO	AMY
	(μmol min^−1^ mL^−1^)											
	37 °C, pH 7	37 °C, pH 4.5	50 °C, pH 7	50 °C, pH 4.5	37°C, pH 7	37°C, pH 4.5	50°C, pH 7	50°C, pH 4.5	37°C, pH 5.0	30°C, pH 4.8	37°C, pH 7.5	30°C, pH 5.0
G61/18	0.75 ± 0.02	0.16 ± 0.01	0.23 ± 0.02	0.29 ± 0.04	0.32 ± 0.01	0.15 ± 0.01	-	-	48.17 ± 0.03	139.56 ± 0.10	-	915.58 ± 0.17
G63/18	0.32 ± 0.03	-	-	-	0.56 ± 0.02	0.08 ± 0.00	0.77 ± 0.08	0.09 ± 0.00	15.85 ± 0.02	616.35 ± 0.06	0.02 ± 0.02	-
G64/18	0.36 ± 0.01	0.10 ± 0.00	1.23 ± 0.16	-	0.40 ± 0.03	0.09 ± 0.00	0.38 ± 0.00	0.53 ± 0.04	63.50 ± 0.16	214.23 ± 0.16	0.69 ± 0.04	585.11 ± 0.30
G65/18	0.15 ± 0.10	-	0.80 ± 0.11	0.29 ± 0.00	0.52 ± 0.02	-		-	85.28 ± 0.06	2750.12 ± 0.08	-	441.53 ± 0.34
G67/18	0.33 ± 0.01	-	0.65 ± 0.02	0.39 ± 0.02	-	-	0.20 ± 0.02	0.48 ± 0.01	38.16 ± 0.13	1294.56 ± 0.16	0.37 ± 0.08	959 ± 0.35
G69/18	-	-	0.03 ± 0.03	0.68 ± 0.02	1.15 ± 0.04	-	0.26 ± 0.06	-	87.05 ± 0.05	-	0.36 ± 0.02	541.73 ± 0.22
G70/18	0.75 ± 0.02	-	-	0.74 ± 0.02	-	-	-	-	254.00 ± 0.07	1708.88 ± 0.41	0.77 ± 0.02	1130.11 ± 0.11
G78/18	-	0.19 ± 0.02	0.87 ± 0.10	0.26 ± 0.01	0.23 ± 0.00	0.13 ± 0.00	-	0.17 ± 0.04	82.98 ± 0.02	141.38 ± 0.08	0.17 ± 0.05	443.40 ± 0.25
G109/18	1.00 ± 0.02	-	0.94 ± 0.02	0.20 ± 0.02	0.02 ± 0.00	-	-	-	23.30 ± 0.01	1367.21 ± 0.10	0.23 ± 0.03	732.40 ± 0.10
G379/18	0.26 ± 0.07	0.39 ± 0.01	0.30 ± 0.05	-	0.42 ± 0.01	-	-	0.40 ± 0.01	26.20 ± 0.02	135.32 ± 0.17	-	-
G398/18	0.13 ± 0.03	0.99 ± 0.04	-	2.02 ± 0.03	0.58 ± 0.02	-	-	-	24.15 ± 0.05	288.83 ± 0.14	0.08 ± 0.00	508.72 ± 0.29

**Table 4:** The early effect of the *Trichoderma* isolates on the nutrient content of the plant (leaves and stem) and soil chemical properties in the *Polana* raspberry pot experiment depended on the pathosystem and naturalization strategy applied, according to pathosystems *Botrytis*, *Verticillium*, *Colletotrichum*, *Phytophthora*, and all pathogens (*Botrytis*–*Verticillium*–*Colletotrichum*–*Phytophthora*) and without pathogens, within different *Trichoderma*-naturalization strategies: no naturalization (NN), root inoculations (R), root inoculations and watering (RW), watering (W).

Pathosystem	Naturalization	Macronutrients in Stems and Leaves					Absorbable Forms of Minerals in Soil			Nitrogen Forms in Soil		
		N	P	K	Ca	Mg	P_2_O_5_	K_2_O	Mg	N-NO_3_	N-NH_4_	N_min_
		% DM					mg/100 g soil			mg/kg DM		kg/ha
Without pathogen	NN	1.44	0.29	2.47	1.22	0.44	18.3	32.5	14.3	4.41	6.26	45.9
	R	1.58	0.30	2.63	1.27	0.46	18.3	32.2	12.8	5.53	4.76	44.2
	RW	1.43	0.26	1.96	1.17	0.44	19.4	31.6	13.7	3.14	4.25	31.8
	W	1.36	0.26	2.33	1.17	0.46	19.5	34.8	12.3	8.91	4.17	56.2
*Botrytis* sp.	NN	1.41	0.28	2.12	1.13	0.46	19.6	35.6	12.6	3.65	7.71	48.8
	R	1.25	0.28	1.96	1.19	0.44	19.7	35.2	11.7	4.59	7.84	53.4
	RW	1.41	0.31	2.47	1.21	0.42	20.2	34.8	13.3	3.10	7.19	44.2
	W	1.21	0.28	1.94	1.12	0.36	16.4	34.8	14.9	3.59	6.32	42.6
*Verticillium* sp.	NN	1.51	0.29	2.04	1.20	0.42	16.1	31.8	14.0	2.23	9.37	49.9
	R	1.28	0.28	1.75	1.10	0.38	15.9	32.5	13.7	1.66	7.46	39.2
	RW	1.53	0.27	1.91	1.20	0.36	16.2	32.9	13.9	1.81	7.33	39.3
	W	1.31	0.27	2.24	1.23	0.36	16.8	31.7	12.3	1.32	9.71	41.8
*Colletotrichum* sp.	NN	1.18	0.26	2.01	1.33	0.38	17.5	31.8	12.8	2.78	7.45	44.0
	R	1.57	0.29	2.56	1.34	0.44	17.1	32.1	12.9	6.68	4.20	46.8
	RW	1.60	0.27	2.42	1.41	0.44	17.8	31.2	12.9	13.37	2.01	66.1
	W	1.08	0.29	2.03	1.26	0.40	20.4	34.5	13.7	3.53	6.96	45.1
*Phytophthora* sp.	NN	1.60	0.28	2.56	1.36	0.40	18.5	32.3	14.4	4.4	6.61	47.3
	R	1.48	0.25	2.35	1.27	0.38	20.0	37.2	14.9	8.76	8.25	73.1
	RW	1.51	0.26	2.00	1.27	0.40	15.3	33.0	14.9	2.12	6.68	37.8
	W	1.51	0.28	2.58	1.34	0.40	17.7	31.3	12.4	7.20	4.81	51.6
All pathogens	NN	1.54	0.27	2.23	1.24	0.39	16.7	30.4	12.9	4.32	9.15	57.9
	R	1.44	0.30	2.22	1.15	0.38	18.7	36.2	14.5	2.30	10.14	53.5
	RW	1.56	0.29	2.12	1.26	0.36	18.9	38.7	13.7	1.84	8.89	46.1
	W	1.14	0.27	2.15	1.29	0.40	16.8	34.0	13.8	2.54	11.53	60.5

**Table 5:** *Trichoderma* isolates sporulation intensity on different microbiological media. Abbreviations: the intensity of the color shown corresponds to the intensity of sporulation—from dark green/green, which reflects excellent sporulation, through to pale cream, reflecting weak sporulation or/and mycelium growth, to white—showing a lack of growth, Potato Dextrose Agar (PDA), Malt Extract Lab Agar (MEA), Corn Meal with glycerol (CM), modified Mandels and Andreotti with agar (MA), soy flour, cellulose, lactose agar medium (MSCL-A), yeast and malt extract minimal agar medium (TR), minimal agar medium with cellulose (MN), wheat bran and apple pomace agar medium with supplements (WBAP-A), wheat bran and apple pomace agar medium with CaCl_2_ and KH_2_PO_4_ (WBAP-B), wheat bran and apple pomace agar medium with microcrystalline cellulose and soy flour (WBAP-C), wheat bran and apple pomace agar medium with microcrystalline cellulose, soy flour, CaCl_2_ and KH_2_PO_4_ (WBAP-D), wheat bran and apple pomace medium (WBAP-E), wheat bran and apple pomace medium with grounded straw and microcrystalline cellulose (WBAP-F), wheat bran, and apple pomace medium with microcrystalline cellulose (WBAP-G), wheat bran and apple pomace medium with pine sawdust (WBAP-H), liquid medium based on soy flour, cellulose, lactose (MSCL-L).

Medium		*Trichoderma* spp.										
		G61/18	G63/18	G64/18	G65/18	G67/18	G69/18	G70/18	G78/18	G109/18	G379/18	G398/18
Agar medium	MSCL-A											
	TR											
	MN											
	MA											
	WBAP-A											
	WBAP-B											
	WBAP-C											
	WBAP-D											
Solid-state medium	WBAP-E											
	WBAP-F											
	WBAP-G											
	WBAP-H											
Liquid and commercial agar medium	MSCL-L											
	PDA											
	CM											
	MEA											
Average spores’ number per plate *		1.31 × 10^10^	1.66 × 10^10^	6.67 × 10^10^	1.14 × 10^10^	6.53 × 10^9^	3.83 × 10^10^	6.61 × 10^10^	1 × 10^10^	3.614 × 10^9^	0	0

* Average spores’ number per plate was calculated using Thoma’s chamber for 90-mm Petri plates with excellent sporulation with respect to each isolate (marked with dark green/green).

**Table 6:** Gelling agent addition screening. The mass of the adhering suspension was measured using raspberry roots weighing 3.93 g.

Gelling Agent Concentration (%)	Water Solution (%)	Xanthan Gum		Citrus Pectin		CarboxymethylCellulose		Sodium Alginate		Guar Gum		Locust Bean Gum		Apple Pectin	
		Adhering Suspension (g)	Viscosity (mPa)	Adhering Suspension (g)	Viscosity (mPa)	Adhering Suspension (g)	Viscosity (mPa)	Adhering Suspension (g)	Viscosity (mPa)	Adhering Suspension (g)	Viscosity (mPa)	Adhering Suspension (g)	Viscosity (mPa)	Adhering Suspension (g)	Viscosity (mPa)
0.5%	1%	<1.00	3.62	<1.00	4.54	<1.00	4.28	<1.00	4.10	<1.00	6.41	<1.00	3.54	-	-
	5%	<1.00	8.10	<1.00	4.98	-	-	-	-	-	-	-	-	-	-
	10%	1.44	9.53	<1.00	5.60	1.36	7.25	<1.00	7.80	<1.00	8.54	<1.00	5.45	-	-
	15%	1.51	21.17	1.14	7.36	-	-	-	-	-	-	-	-	-	-
	20%	1.91	32.01	1.42	9.09	1.81	10.82	1.32	11.04	1.02	9.82	<1.00	7.80	-	-
1%	1%	<1.00	4.54	<1.00	4.32	-	-	-	-	-	-	-	-	-	-
	5%	1.22	8.76	<1.00	5.16	-	-	-	-	-	-	-	-	-	-
	10%	1.70	14.19	<1.00	6.37	-	-	-	-	-	-	-	-	-	-
	15%	2.03	32.12	<1.00	8.36	-	-	-	-	-	-	-	-	-	-
	20%	2.68	53.38	1.10	9.93	-	-	-	-	-	-	-	-	-	-
2%	1%	<1.00	6.04	<1.00	5.34	<1.00	5.71	<1.00	6.78	<1.00	4.94	<1.00	4.06	-	-
	5%	1.88	16.36	<1.00	6.11	-	-	-	-	-	-	-	-	-	-
	10%	2.13	32.96	<1.00	7.33	<1.00	9.64	1.23	12.98	<1.00	9.13	<1.00	7.44	-	-
	15%	3.21	65.61	<1.00	9.64	-	-	-	-	-	-	-	-	-	-
	20%	5.42	116.8	1.38	14.23	1.11	29.51	1.72	55.99	1.51	28.3	1.34	10.41	-	-
4%	1%	<1.00	8.43	<1.00	4.72	-	-	-	-	-	-	-	-	-	-
	5%	1.66	28.37	<1.00	6.34	-	-	-	-	-	-	-	-	-	-
	10%	3.79	75.51	<1.00	8.83	-	-	-	-	-	-	-	-	-	-
	15%	6.71	144.50	<1.00	15.11	-	-	-	-	-	-	-	-	-	-
	20%	7.90	177.90	2.31	36.74	-	-	-	-	-	-	-	-	-	-
6%	1%	1.07	9.13	<1.00	5.67	<1.00	7.25	<1.00	9.02	<1.00	<1.00	<1.00	3.76	-	-
	5%	2.40	45.56	<1.00	7.73	-	-	-	-	-	-	-	-	-	-
	10%	3.25	103.30	<1.00	11.99	1.61	30.06	1.86	54.63	3.37	72.33	2.56	11.62	-	-
	15%	7.60	195.30	2.89	30.13	-	-	-	-	-	-	-	-	-	-
	20%	10.14	335.70	3.41	65.53	2.68	181.40	4.32	>365.00	7.23	238.60	3.76	60.61	-	-
15%	20%	-	-	5.24	>365.00	-	-	-	-	-	-	-	-	3.90	172.50
20%	15%	-	-	6.21	353.80	-	-	-	-	-	-	-	-	2.98	168.20

## Data Availability

Data is contained within the article or Supplementary Materials.

## Supplementary Materials

The following are available online at https://www.mdpi.com/article/10.3390/ijms22126356/s1.

## Abbreviations

AMY, amylase activity; BGL, β-glucosidase activity; CM, Corn Meal agar medium with glycerol; CMC carboxymethylcellulases activity; D-GE, gel formulation; D-PW, dissolvable formulation; FF, filamentous fungi; FPU cellulases activity; IA PAS, Institute of Agrophysics, Polish Academy of Sciences; MA, modified Mandels and Andreotti agar medium; MEA, Malt Extract Lab Agar medium; MN, minimal agar medium with cellulose; MSCL-A, soy flour, cellulose, lactose agar medium; MSCL-L, soy flour, cellulose, lactose liquid medium; NN, no naturalization system; PDA, Potato Dextrose Lab agar medium; PRO, protease activity; R, root inoculations system; RW, root inoculations and watering system; SOM, soil organic matter; S-PE, pelleting formulation; S-PW, powder formulation; TDR, time-domain reflectometry; TR, yeast and malt extract minimal agar medium; W, watering system; WBAP, solid state medium based on wheat bran and apple pomace and whey protein; WBAP-A, agar medium based on wheat bran, apple pomace and whey protein with supplements; WBAP-B, agar medium based on wheat bran, apple pomace and whey protein with CaCl2 and KH2PO4; WBAP-C, agar medium based on wheat bran, apple pomace and whey protein with microcrystalline cellulose and soy flour; WBAP-D, agar medium based on wheat bran, apple pomace and whey protein with microcrystalline cellulose, soy flour, CaCl2 and KH2PO4; WBAP-E, solid-state medium based on wheat bran, apple pomace and whey protein; WBAP-F, solid-state medium based on wheat bran, apple pomace and whey protein with straw and microcrystalline cellulose; WBAP-G, solid-state medium based on wheat bran, apple pomace and whey protein with microcrystalline cellulose; WBAP-H, solid-state medium based on wheat bran, apple pomace and whey protein with sawdust; WPC, whey protein concentrate; XYL, xylanase activity.
