# Supplementary material for: Trichoderma-Based Biopreparation with Prebiotics Supplementation for the Naturalization of Raspberry Plant Rhizosphere
Source: Int J Mol Sci. 2021 Jun 14;22(12):6356. doi: 10.3390/ijms22126356 (PMC8232080; doi:10.3390/ijms22126356)
Supplement: Supplementary file 1 [file ijms-22-06356-s001.zip › Table S1.pdf]

International Journal of *Molecular Sciences*

MDPI

Table S1. The antagonistic properties of wild raspberry fungal isolates against phytopathogens *Colletotrichum* spp. (including *C. acutatum*), *Verticillium* spp., *Phytophthora* spp., and *Botrytis* spp. (including *B. cinerea*), resulting in growth and/or sporulation inhibition in an experiment on Petri dishes. Abbreviations: the antagonism abilities rating was used: "+++, ++, +, -" meaning excellent, very good, good, and no response, respectively.

	Colletotrichum spp.			*Verticillium* spp.			*Phytophthora* spp.			*Botrytis* spp.		
**Isolate**	**G172/18**	**G371/18**	**G166/18**	**G293/18**	**G296/18**	**G297/18**	**G368/18**	**G373/18**	**G369/18**	**G275/18**	**G277/18**	**G276/18**
G27/18	-	-	-	-	-	-	-	-	-	-	-	-
G28/18	-	-	-	-	-	-	-	-	-	-	-	-
G29/18	-	-	-	-	-	-	-	-	-	-	-	-
G40/18	-	-	-	-	-	-	-	-	-	-	-	-
G43/18	-	-	-	-	-	-	-	-	-	-	-	-
G48/18	-	-	+	-	-	-	-	-	-	-	-	-
G56/18	-	-	-	-	-	-	-	-	-	-	+	+
G57/18	-	-	-	-	+	-	+	-	+	-	-	-
G58/18	-	-	-	-	-	-	+	+	-	-	-	+
G59/18	+	++	+	+	+	+	++	+	++	++	++	+++
G60/18	++	++	++	++	++	++	+	+	++	++	+	++
G61/18	++	++	+	++	++	+++	++	+	+	+	++	++
G62/18	+	++	++	+	++	+	+	+	+	++	+++	++
G63/18	+	++	++	++	++	++	+	++	++	++	++	++
G64/18	+	++	+++	++	++	++	++	++	++	++	++	++
G65/18	++	+	+	++	++	++	++	+	++	-	+	+
G66/18	++	+	+	++	++	++	++	+	++	+	+	+
G67/18	+	+	+	++	++	++	++	++	++	+	+	+
G68/18	++	++	++	++	++	++	++	+	++	++	++	+
G69/18	++	+	+	++	++	++	++	+	++	++	++	++
G70/18	+	+	+	+	+	+	+++	++	+++	+	+	+
G71/18	++	++	++	++	++	++	++	+	++	+	+	+
G72/18	++	++	++	+++	++	++	++	++	+++	++	++	++
G73/18	++	++	++	++	++	+++	+	++	++	++	++	++
G74/18	+	++	+	++	+++	+++	++	++	++	++	++	++
G75/18	+	+	+	++	++	++	+	++	++	++	++	++
G76/18	++	++	++	++	++	++	++	+	++	-	+	+
G77/18	+	+	++	++	++	+	++	+	++	+	+	+
G78/18	+	+	+	++	++	++	++	+	++	-	+	+
G79/18	+	+	++	++	++	+++	+	+	++	+	+	+
G80/18	+	++	+	++	++	++	-	+	++	+	++	+++

International Journal of
Molecular Sciences

MDPI

G86/18	-	-	-	+	+	+	+	+	+	-	-	+
G87/18	-	-	-	+	+	+	++	+	++	+	++	++
G90/18	-	-	-	+	++	+	+++	++	++	++	+	++
G92/18	+	-	-	+	+	+	++	+	++	+	+	+
G97/18	++	++	++	+	++	++	-	+	++	+	+	++
G98/18	-	-	-	-	-	-	-	-	-	-	-	-
G99/18	-	-	-	-	-	-	-	-	-	-	-	-
G100/18	-	-	-	-	-	-	-	-	-	-	-	-
G101/18	-	-	-	+	-	-	-	-	-	-	-	-
G102/18	-	-	-	-	-	-	-	-	-	-	-	-
G103/18	-	-	-	-	+	-	-	-	-	-	-	+
G104/18	-	-	-	-	-	-	-	-	-	-	-	-
G105/18	-	-	-	-	-	+	-	-	-	-	-	-
G109/18	+	+	+	++	+	+	++	++	+++	+	+	+
G111/18	-	-	-	+	+	+	-	+	-	+	-	+
G112/18	-	+	-	-	-	-	+	+	+	+	+	++
G113/18	+	+	++	-	+	-	+	+	-	++	+	+
G114/18	-	-	+	-	+	-	-	+	+	+	++	+++
G118/18	++	++	++	-	+	++	++	+++	+	+	+	+
G119/18	++	++	+	+	++	++	+++	++	++	++	+	+
G126/18	-	+	+	-	+	+	-	+++	-	+++	+++	+++
G132/18	-	-	-	-	-	-	-	-	-	-	-	-
G133/18	-	-	-	-	-	-	-	-	-	-	-	-
G134/18	-	-	-	-	-	-	-	-	-	-	-	-
G135/18	-	-	-	-	-	-	-	-	-	-	-	-
G137/18	-	-	-	-	-	-	-	-	-	-	-	-
G138/18	-	-	-	-	-	-	-	-	-	-	-	-
G139/18	+	+	-	-	-	-	-	+	-	+++	+++	++
G140/18	-	-	-	-	+	++	+	++	+	+	-	-
G141/18	-	+	-	-	-	+	-	++	++	+	+	+
G143/18	-	-	-	-	+	+	-	+++	+++	+	-	++
G149/18	-	-	-	-	-	-	-	-	-	-	-	-
G150/18	-	-	-	-	-	-	-	-	-	-	-	-
G152/18	-	-	-	-	-	-	-	+	-	-	-	-
G153/18	-	-	-	-	-	-	-	-	-	-	-	+
G155/18	-	-	-	+	++	+	+	+	+	-	-	-
G158/18	-	-	-	-	+	+	-	++	++	-	-	-
G159/18	-	-	-	-	+	+	+	++	++	-	-	+

International Journal of Molecular Sciences

MDPI

G375/18	+	-	-	+	+	+	++	-	++	-	+	+
G376/18	-	-	-	-	-	-	-	+	-	-	+	-
G377/18	-	-	-	-	-	-	-	-	-	-	-	-
G378/18	+	++	++	++	+	++	++	++	++	+	+	+
G379/18	-	+	+	+	+	++	-	++	++	++	+	+
G380/18	-	-	-	-	-	-	-	-	+	-	-	-
G381/18	-	-	-	-	+	-	+	+	++	-	-	-
G382/18	-	-	-	-	-	-	-	+	-	-	-	-
G383/18	-	-	-	+	++	+	++	+	+++	++	+	++
G384/18	-	-	-	+	+	-	++	-	++	-	-	-
G385/18	-	-	-	+	-	+	++	+++	+++	++	++	++
G386/18	-	-	-	+	+	+	+	+	++	-	-	-
G387/18	+	+++	+	+++	++	+++	++	+	+++	++	+	+++
G388/18	++	++	++	+	-	++	++	++	++	++	+	+
G389/18	+	-	-	+	+	++	++	++	++	+	+	++
G390/18	-	-	-	-	-	-	++	++	+++	-	-	+
G391/18	-	+	+	-	-	+	++	+++	++	++	++	++
G392/18	++	++	++	+	+	+	++	++	+	+	+	+
G393/18	+	++	++	-	-	++	++	++	++	+	+	+
G394/18	+	-	+	-	-	+	-	+	+	+	+	-
G395/18	-	-	-	-	+	+	+	+	+	-	+	-
G396/18	-	-	-	+	-	-	+++	++	+++	+	+	++
G39718	-	-	-	+	+	+	+	+	++	+	-	-
G398/18	-	+	+	++	++	+++	++	++	++	+	+	+
G399/18	-	++	+	+	+	++	++	+	++	+	+	+
