# Supplementary material for: Trichoderma-Based Biopreparation with Prebiotics Supplementation for the Naturalization of Raspberry Plant Rhizosphere
Source: Int J Mol Sci. 2021 Jun 14;22(12):6356. doi: 10.3390/ijms22126356 (PMC8232080; doi:10.3390/ijms22126356)
Supplement: Supplementary file 1 [file ijms-22-06356-s001.zip › Table S2.pdf]

Table S2. The data set of culture media composition and the conditions used in screening for *Trichoderma* isolates sporulation. Abbreviations: Potato Dextrose Agar (PDA), Malt Extract Lab Agar (MEA), Corn Meal with glycerol (CM), modified Mandels and Andreotti with agar (MA), soy flour, cellulose, lactose agar medium (MSCL-A), yeast and malt extract minimal agar medium (TR), minimal agar medium with cellulose (MN), wheat bran and apple pomace agar medium with supplements (WBAP-A), wheat bran and apple pomace agar medium with $CaCl_2$ and KH_2PO_4 (WBAP-B), wheat bran and apple pomace agar medium with microcrystalline cellulose and soy flour (WBAP-C), wheat bran and apple pomace agar medium with microcrystalline cellulose, soy flour, $CaCl_2$ and KH_2PO_4 (WBAP-D), wheat bran and apple pomace medium (WBAP-E), wheat bran and apple pomace medium with grounded straw and microcrystalline cellulose (WBAP-F), wheat bran, and apple pomace medium with microcrystalline cellulose (WBAP-G), wheat bran and apple pomace medium with pine sawdust (WBAP-H), liquid medium based on soy flour, cellulose, lactose (MSCL-L).

Agar medium							
**MSCL-A**		**TR**		**MN**		**MA**	
Lactose	17.5 g/l	Glucose	10 g/l	$(NH_4)_2SO_4$	3 g/l	$(NH_4)_2SO_4$	1.4 g/l
Microcrystalline cellulose	11.8 g/l	KH_2PO_4	11 g/l	KH_2PO_4	1 g/l	KH_2PO_4	2.0 g/l
Soy flour	17.5 g/l	Yeast extract	5 g/l	$MgSO_4$ x $7H_2O$	5 g/l	$MgSO_4$ x $7H_2O$	0.3 g/l
KH_2PO_4	6.3 g/l	Malt extract	30 g/l	Yeast extract	5 g/l	$CaCl_2$ x $2H_2O$	0.3 g/l
$(NH_4)_2SO_4$	9.92 g/l	Agar	20 g/l	Microcrystalline cellulose	20 g/l	Urea	0.3 g/l
$CaCl_2$	1.64 g/l	pH	5	Agar	20 g/l	Casein peptone	1.0 g/l
$MgSO_4$ x $7H_2O$	0.82 g/l	Spores inoculum	10^2	pH	5	Glucose	10 g/l
Tween 80	0.15%	Temperature	26°C	Spores inoculum	10^2	Micronutrient solution	20 ml/l
Antifoam B	5 ml/l			Temperature	26°C	$FeSO_4$ x $7H_2O$	250 mg/l
Micronutrient solution	20 ml/l					$MnSO_4$ x H_2O	80 mg/l
$FeSO_4$ x $7H_2O$	513 mg/l					$ZnSO_4$ x $7H_2O$	70 mg/l
$MnSO_4$ x H_2O	166 mg/l					Agar	20 g/l
$ZnSO_4$ x 7H2O	8.5 mg/l					pH	5
$CoCl_2$ x $6H_2O$	204 mg/l					Spores inoculum	10^2
Agar	35 g/l					Temperature	26°C
pH	5						
Spores inoculum	10^2						
Temperature	26°C						
**WBAP-A**		**WBAP-B**		**WBAP-C**		**WBAP-D**	
Wheat bran Durum	7 g/l	Wheat bran Durum	7 g/l	Wheat bran Durum	7 g/l	Wheat bran Durum	7 g/l
Dried apple pomace	14 g/l	Dried apple pomace	14 g/l	Dried apple pomace	14 g/l	Dried apple pomace	14 g/l
Whey protein concentrate	5 g/l	Whey protein concentrate	5 g/l	Whey protein concentrate	5 g/l	Whey protein concentrate	5 g/l
Saccharose	1 g/l	Saccharose	1 g/l	Saccharose	1 g/l	Saccharose	1 g/l

Glucose	1 g/l	Glucose	1 g/l	Glucose	1 g/l	Glucose	1 g/l
$(NH_4)_2HPO_4$ 1%	15 ml/l	$(NH_4)_2HPO_4$ 1%	15 ml/l	$(NH_4)_2HPO_4$ 1%	15 ml/l	$(NH_4)_2HPO_4$ 1%	15 ml/l
Micronutrient solution	10 ml/l	$CaCl_2$	1.6 g/l	Microcrystalline cellulose	11.67 g/l	$CaCl_2$	1.6 g/l
$FeSO_4 \times 7H_2O$	250 mg/l	KH_2PO_4	6.3 g/l	Soy flour	17.5 g/l	KH_2PO_4	6.3 g/l
$MnSO_4 \times H_2O$	80 mg/l	Micronutrient solution	10 ml/l	Micronutrient solution	10 ml/l	Soy flour	17.5 g/l
$ZnSO_4 \times 7H_2O$	70 mg/l	$FeSO_4 \times 7H_2O$	250 mg/l	$FeSO_4 \times 7H_2O$	250 mg/l	Microcrystalline cellulose	11.67 g/l
Adenosine	1.6 g/l	$MnSO_4 \times H_2O$	80 mg/l	$MnSO_4 \times H_2O$	80 mg/l	Micronutrient solution	10 ml/l
Adonitol	1.6 g/l	$ZnSO_4 \times 7H_2O$	70 mg/l	$ZnSO_4 \times 7H_2O$	70 mg/l	$FeSO_4 \times 7H_2O$	250 mg/l
Arabitol	1.6 g/l	Agar	35 g/l	Agar	35 g/l	$MnSO_4 \times H_2O$	80 mg/l
Erythritol	1.6 g/l	pH	5	pH	5	$ZnSO_4 \times 7H_2O$	70 mg/l
Mannitol	1.6 g/l	Spores inoculum	10^2	Spores inoculum	10^2	Agar	35 g/l
Sorbitol	1.6 g/l	Temperature	26°C	Temperature	26°C	pH	5
Agar	35 g/l					Spores inoculum	10^2
pH	5					Temperature	26°C
Spores inoculum	10^2						
Temperature	26°C						
**SOLID-STATE MEDIUM**							
**WBAP-E**		**WBAP-F**		**WBAP-G**		**WBAP-H**	
Plastic transparent bag	50 cm x 70 cm	Conical flask	500 ml	Conical flask	250 ml	Conical flask	250 ml
Wheat bran Durum	70 g	Wheat bran Durum	3.5 g	Wheat bran Durum	1.75 g	Wheat bran Durum	1.75 g
Dried apple pomace	14 g	Dried apple pomace	7 g	Dried apple pomace	3.5 g	Dried apple pomace	3.5 g
Whey protein concentrate	50 g	Whey protein concentrate	2.5 g	Whey protein concentrate	1.25 g	Whey protein concentrate	1.25 g
Micronutrient solution	100 ml	Grounded straw	1 g	Microcrystalline cellulose	0.41g	Pine sawdust	0.5 g
$FeSO_4 \times 7H_2O$	250 mg/l	Microcrystalline cellulose	0.275 g	Micronutrient solution	2.5 ml/l	Micronutrient solution	2.5 ml/l
$MnSO_4 \times H_2O$	80 mg/l	Micronutrient solution	5 ml/l	$FeSO_4 \times 7H_2O$	250 mg/l	$FeSO_4 \times 7H_2O$	250 mg/l
$ZnSO_4 \times 7H_2O$	70 mg/l	$FeSO_4 \times 7H_2O$	250 mg/l	MnSO4 x H2O	80 mg/l	MnSO4 x H2O	80 mg/l
$(NH_4)_2HPO_4$	1.5 g	$MnSO_4 \times H_2O$	80 mg/l	ZnSO4 x 7H2O	70 mg/l	ZnSO4 x 7H2O	70 mg/l
Saccharose	10 g	$ZnSO_4 \times 7H_2O$	70 mg/l	$(NH_4)_2HPO_4$	3.75 g	$(NH_4)_2HPO_4$	3.75 g
Glucose	10 g	$(NH_4)_2HPO_4$	7.5 g	Saccharose	0.25 g	Saccharose	0.25 g
Spores inoculum 70%T	50 ml	Saccharose	0.5 g	Glucose	0.25 g	Glucose	0.25 g
Temperature	22-26°C	Glucose	0.5 g	Spores inoculum	10^3	Spores inoculum	10^3

		Spores inoculum	10^3	Temperature	26°C	Temperature	26°C
		Temperature	26°C				
**LIQUID & COMMERCIAL AGAR MEDIUM**							
**MSCL-L**		**PDA**		**CM**		**MEA**	
Conical flask / Medium volume	100 ml / 50 ml						
Lactose	17.5 g/l						
Microcrystalline cellulose	11.8 g/l	Oxoid®	39 g/l	Biomaxima®	17 g/l	Biocorp®	33 g/l
Soy flour	17.5 g/l	Spores inoculum	10^2	Spores inoculum	10^2	Spores inoculum	10^2
KH_2PO_4	6.3 g/l	Temperature	26°C	Temperature	26°C	Temperature	26°C
$(NH_4)_2SO_4$	9.92 g/l						
$CaCl_2$	1.64 g/l						
$MgSO_4$ x $7H_2O$	0.82 g/l						
Tween 80	0.15%						
Antifoam B	5 ml/l						
Micronutrient solution	20 ml/l						
FeSO4 x 7H2O	513 mg/l						
MnSO4 x H2O	166 mg/l						
$ZnSO_4$ x 7H2O	8.5 mg/l						
$CoCl_2$ x $6H_2O$	204 mg/l						
pH	5						
rpm	150						
Spores inoculum	10^3						
Temperature	26°C						
